# In Situ Metabolic Rates of Alkane‐Degrading Sulphate‐Reducing Bacteria in Hydrocarbon Seep Sediments Revealed by Combining CARD‐FISH, NanoSIMS, and Mathematical Modelling

**DOI:** 10.1111/1462-2920.70151

**Published:** 2025-07-29

**Authors:** Sara Kleindienst, Lubos Polerecky, Rudolf Amann, Florin Musat, Katrin Knittel

**Affiliations:** ^1^ Max Planck Institute for Marine Microbiology Bremen Germany; ^2^ Department of Environmental Microbiology, Institute for Sanitary Engineering, Water Quality and Solid Waste Management (ISWA) University of Stuttgart Stuttgart Germany; ^3^ Department of Earth Sciences, Faculty of Geosciences Utrecht University Utrecht the Netherlands; ^4^ Department of Biology, Section for Microbiology Aarhus University Aarhus Denmark

**Keywords:** activity, alkane degraders, anaerobic butane degradation, anaerobic dodecane degradation, cell numbers, hydrocarbon seeps, marine sediments, NanoSIMS, stable‐isotope probing

## Abstract

Marine hydrocarbon seeps are hotspots for sulphate reduction coupled to hydrocarbon oxidation. In situ metabolic rates of sulphate‐reducing bacteria (SRB) degrading hydrocarbons other than methane, however, remain poorly understood. Here, we assessed the environmental role of *Desulfosarcinaceae* clades SCA1, SCA2 for degradation of *n*‐butane and clade LCA2 for *n*‐dodecane. Quantification by CARD‐FISH showed that SCA1 constituted up to 31%, SCA2 up to 9%, and LCA2 up to 6% of cells from the recently re‐classified class *Deltaproteobacteria* across diverse hydrocarbon seeps. Cell‐specific oxidation rates estimated by stable‐isotope probing combined with NanoSIMS and modelling were ~0.73 and ~2.11 fmol butane cell^−1^ d^−1^ for SCA1 and SCA2, respectively, and ~0.023 fmol dodecane cell^−1^ d^−1^ for LCA2 in sediments from Amon Mud Volcano and Guaymas Basin sediments. Cellular carbon assimilation, dissolved inorganic carbon production, and sulphate reduction rates indicated that butane‐degrading SRB have higher metabolic activity than those utilising dodecane. Estimates based on in situ cell abundances, biovolumes, and cellular activities suggest that at certain seeps, clades SCA1, SCA2 and LCA2 account for nearly all sulphate reduction not driven by methane oxidation. These findings highlight the important role of alkane‐degrading SRB in influencing marine carbon and sulphur cycles, particularly at seeps emitting higher hydrocarbons.

## Graphical abstract

Sulphate‐reducing bacteria affiliated with clades SCA1, SCA2, and LCA2 were investigated using stable‐isotope incubations and in situ analysis at several hydrocarbon seep sites. Extrapolated rates of sulphate reduction and alkane degradation suggest that these clades play an important role at hydrocarbon seeps, contributing substantially to sulphur and carbon cycling.

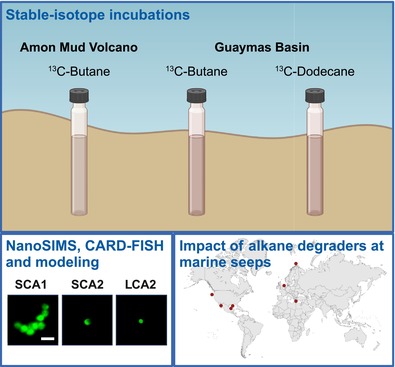

## 1 Introduction

Hydrocarbon seepage in the form of gas and oil is widespread in the marine environment (e.g., Byrne and Emery 1960; Simoneit and Lonsdale 1982; Anderson et al. 1983; Kennicutt II et al. 1988; Mastalerz et al. 2009). In sediments impacted by hydrocarbon seepage, rates of sulphate reduction (SR) are in the millimolar per day range and therefore several orders of magnitude higher compared to those in non‐seep sediments (Aharon and Fu 2000; Boetius et al. 2000; Michaelis et al. 2002; Treude et al. 2003; Joye et al. 2004). At methane‐dominated seeps, SR is mainly fuelled by the anaerobic oxidation of methane (AOM). However, the global median SR rates at seeps exceed AOM rates about 10‐fold (Bowles et al. 2011), indicating sulphate‐dependent oxidation of hydrocarbons other than methane as a major process at marine seeps. Indeed, at seeps with emissions of complex hydrocarbon mixtures, several biogeochemical studies showed the importance of microbial oxidation of non‐methane hydrocarbons by sulphate‐reducing bacteria (SRB) of *Desulfobacterota* (Joye et al. 2004; Kallmeyer and Boetius 2004; Mastalerz et al. 2009; Omoregie et al. 2009; Orcutt et al. 2010; Bowles et al. 2011; Singh et al. 2017). Recent studies have also uncovered numerous predominantly anaerobic archaeal clades that are able to use higher alkanes by alkyl‐coenzyme M reductases (for review, see Musat et al. 2024). With the exception of *Ca*. Methanoliparia archaea, which couple the oxidation of C13‐C39 alkanes with methane formation (Zhou et al. 2022), and of *Ca*. Melinoarchaeum, a C16 alkane degrader growing in syntrophy with methanogens (Yu et al. 2024), all other known anaerobic hydrocarbon‐degrading archaea live in syntrophy with SRB. These include the short‐chain alkane oxidizers *Ca*. Ethanoperedens (Hahn et al. 2020), *Ca*. Argoarchaeum (Chen et al. 2019) and *Ca*. Syntrophoarchaeum (Laso‐Pérez et al. 2016), and the medium‐ to long‐chain alkane oxidizers *Ca*. Alkanophaga (Zehnle et al. 2023).

Community analyses showed that *Desulfobacterota*, and in particular members of the *Desulfosarcina/Desulfococcus* (DSS) clade, are highly diverse and globally abundant in hydrocarbon seep sediments (e.g., Teske et al. 2002; Knittel et al. 2003; Orcutt et al. 2010; Kleindienst et al. 2012; Stagars et al. 2017). Within the DSS clade, four *Desulfosarcinaceae* clades were identified as active alkane degraders in complex benthic communities (Figure S1) (Kleindienst et al. 2014). The butane degraders belonged to two distinct groups, referred to as short‐chain alkane 1 (SCA1) and short‐chain alkane 2 (SCA2). SCA1 comprises numerous 16S rRNA gene sequences derived from several propane‐ and butane‐degrading enrichment cultures (Butane12‐GMe, Propane12‐GMe, Butane12‐HR; Jaekel et al. 2013) as well as from the butane‐degrading sulphate‐reducing bacterium *Desulfosarcina aeriophaga* strain BuS5 (Kniemeyer et al. 2007; Chen et al. 2022). The clade likely encompasses additional, as yet uncultivated genera, as suggested by the 94%–100% intragroup 16S rRNA gene identity. Clade SCA2 comprises only sequences from organisms that have not yet been cultivated, and is defined at the species level (threshold 98.7%, Yarza et al. 2014). The most closely related cultivated organism is *Desulfosudis oleivorans* strain HxD3, with 87% 16S rRNA gene similarity. The identified *n*‐dodecane degraders form two distinct clades, referred to as long‐chain alkane 1 (LCA1) and long‐chain alkane 2 (LCA2), both of which consist exclusively of sequences from uncultivated organisms (Kleindienst et al. 2014). The closest cultivated relative of LCA1 is *Desulfatitalea tepidiphila* (91% 16S rRNA gene similarity), while LCA2 is most closely related to *Desulfatirhabdium butyrativorans* (91% similarity).

Current knowledge on the cellular activities of bacterial short‐chain alkane degraders is primarily derived from sediment‐free enrichment cultures comprising members of the SCA1 clade (Jaekel et al. 2013), but no data are available on these communities in natural sediments. Here we addressed this knowledge gap by investigating the in situ cellular alkane consumption rates of specific alkane‐degrading SRB in sediment incubations, focusing on members of clades SCA1, SCA2, and LCA2. We hypothesise that these specific SRB clades are widespread in seep environments and significantly contribute to alkane removal and sulphate reduction in these habitats.

To test this hypothesis, we amended sediments from two distinct marine hydrocarbon seep sites, the Amon Mud Volcano (Amon MV) in the Mediterranean Sea and the Guaymas Basin in the Gulf of California, with either ^13^C labelled butane or ^13^C labelled dodecane, and incubated them under anoxic conditions at temperatures closely resembling those of their natural environments. We then employed catalysed‐reporter deposition fluorescence in situ hybridisation (CARD‐FISH) for cell identification and quantification, nanometre scale secondary ion mass spectrometry (NanoSIMS) for single‐cell measurements of ^13^C‐uptake, and mathematical modelling to estimate the cellular rates of alkane removal and sulphate reduction of the target groups. We further quantified the in situ abundance of clades SCA1, SCA2, and LCA2 in globally distributed hydrocarbon seep sediments and extrapolated the cellular rates to assess the contribution of these bacterial groups to alkane removal and sulphate reduction at other hydrocarbon seep habitats.

## 2 Experimental Procedures

### 2.1 Sample Collection

For slurry incubations, anoxic sediments from two different seep sites were collected in 2009: (i) Mediterranean Amon MV of the Nile Deep Sea Fan samples were collected during the cruise MSM13‐3 (RV Maria S. Merian; ROVQUEST4000, MARUM) from 2 to 20 cm below a microbial mat (PANGAEA‐EventLabel MSM13/3_929‐1_PUC1; MSM13/3_929‐1_PUC9, MSM13/3_929‐1_PUC20; water depth 1122 m, 32°20.1321′ N, 31°42.6543′ E). A detailed description of the sampling site can be found elsewhere (Grünke et al. 2011). The sediments are characterised by the massive presence of gaseous hydrocarbons. (ii) Guaymas Basin samples were collected during the cruise AT 15–56 (RV Atlantis). The Guaymas Basin in the central Gulf of California harbours petroleum‐rich hydrothermal sediments, covered with organic‐rich layers of buried sedimentary organic matter. Hydrothermal fluids contain remarkable concentrations of hydrocarbons including alkanes and aromatic hydrocarbons (Didyk and Simoneit 1989). Fine‐grained sediment samples below a white *Beggiatoa* mat from 0 to 10 cm sediment depth (push cores 9 and 10, water depth 2010 m, 27°0.696′ N, 111°24.265′ W) with a conspicuous hydrocarbon smell were collected during dive 4573 with the submersible Alvin. Sediments were transferred to Duran bottles and maintained under an anoxic atmosphere (N_2_/CO_2_ 9:1 v/v) for transport and storage for 4 months at 4°C. During this time, preincubations were carried out to identify sediments with the highest alkane‐degrading microbial activities based on sulphide and ^13^CO_2_ production. No decrease in microbial activity was observed between preincubations that were performed shortly after sampling and those in the experiments presented here. Therefore, we do not expect any significant loss in sample quality during the 4 months of storage.

Additionally, we sampled sediments from eight seep sites to study cell abundance and cell size of target groups using CARD‐FISH. Five habitats—Haakon Mosby mud volcano, Amon MV, Hydrate Ridge, Black Sea, and Tommeliten—are characterised by the presence of gaseous hydrocarbons and are referred to as gas seeps. Three habitats—Gulf of Mexico, Guaymas Basin, and Chapopote Asphalt Volcano—are characterised by seepage of complex hydrocarbon mixtures and are referred to as hydrocarbon seeps. Detailed site descriptions can be found in Kleindienst et al. (2012). For each sample, 0.5 g of sediments was directly fixed after sampling in 3% formaldehyde for 2–8 h at 4°C, washed in PBS/ethanol, and finally stored in PBS/ethanol at −20°C.

### 2.2 Experimental Set‐Up

Incubations under sulphate‐reducing conditions were set up with sediments from Amon MV with butane as substrate at 20°C and with sediments from Guaymas Basin with butane or dodecane as substrate at 28°C. Butane is a natural substrate at both seeps occurring at concentrations of 0.5–1.1 μM (Amon MV) and 6–16 μM (Guaymas Basin, at a site close by; M. Kellermann, personal communication). Dodecane is typically found at hydrocarbon seeps from Guaymas Basin (cf., Bazylinski et al. 1988).

For each site, substrate and time point, sediment slurries were prepared under anoxic atmosphere (N_2_/CO_2_ 9:1 v/v) in a 1:1 (v/v) mixture with artificial anoxic seawater buffered with 30 mM bicarbonate (Widdel and Bak 1992). Fully ^13^C‐labelled *n*‐butane or *n*‐dodecane (Campro Scientific, Germany) were added to the slurries at a concentration of 2.1 and 1.8 mM, respectively. These concentrations exceeded the natural concentrations by over 100‐fold to prevent substrate‐diffusion limitations. The sediment slurry was distributed in 4 mL aliquots in 5.7 mL glass vials sealed with butyl rubber stoppers under a headspace of N_2_/CO_2_ 9:1 v/v.

The incubations were followed by sampling at three to six time points. Because sampled vials were sacrificed, samples collected at different time points correspond to different replicate vials. Seven replicate vials were incubated for Amon MV‐butane (*t* = 0, 1, 2, 4, 9, 15, 29 days), and five vials each for Guaymas Basin‐butane (0, 29, 57, 71, 113 days) and Guaymas Basin‐dodecane (0, 29, 115, 183, 232 days). For each time point and sample, the slurries were fixed for 1 h at 4°C with 1% formaldehyde (final concentration), washed with 1× phosphate‐buffered saline (PBS, 10 mM sodium phosphate, 130 mM NaCl, pH 7.3), and stored in 6 mL of ethanol/PBS mixture (1:1 v/v) at –20°C. The fixed slurries were used for TOC analysis, Nano‐SIMS and CARD‐FISH.

We regard the independent vials with the same source material as biological replicates. Since hydrocarbon consumption, DOC, and TOC curves from independent incubations behaved as expected for a continuous incubation, we regard the data as a time series.

### 2.3 Bulk Analyses of ^13^C‐TOC and ^13^C‐DIC

Assimilation of the ^13^C‐labelled substrates was confirmed by bulk analysis of the ^13^C and ^12^C content in the total organic carbon (TOC) pool. To this end, 500 μL of fixed sediment sample was decarbonised using 1 M HCl, dried, and packed into tin‐cups. Analysis was performed with an automated elemental analyser (Thermo Flash EA, 1112 Series, Thermo Fischer, Dreieich, Germany) and a Finnigan Delta Plus Advantage mass spectrometer (Thermo Fischer), using CO_2_ released by flash combustion in excess oxygen at 1050°C. The ^13^C enrichment of the DIC pool was taken from Kleindienst et al. (2014).

### 2.4 Chemical Analyses

Hydrocarbon consumption was either directly measured (butane incubations) or inferred based on sulphide production (dodecane incubations). Sulphide was quantified photometrically as colloidal CuS (Cord‐Ruwisch 1985). For each time point, two biological replicates with two technical replicates each were measured. Butane concentrations were measured by gas‐chromatographic headspace analysis (oven 110°C, injector 150°C, detector 280°C, nitrogen carrier gas) as described before (Musat and Widdel 2008; Kleindienst et al. 2014). Data were published previously in Kleindienst et al. (2014).

### 2.5 Total Cell Counts

The total number of cells was determined in the time course of the incubations by applying acridine orange direct counts (AODC) according to Meyer‐Reil (1983) as modified by Boetius and Lochte (1996). Microscopy was done with an epifluorescence microscope (Nikon eclipse 50i, Düsseldorf, Germany). At least 25 randomly chosen fields of view were counted.

### 2.6 Catalysed Reporter Deposition Fluorescence In Situ Hybridisation (CARD‐FISH)

In addition to the incubations, sediments from eight different seep sites were selected for in situ quantification of the target groups SCA1, SCA2, and LCA2. For these samples as well as the incubations, cells were detached from sediment particles by sonication for 20 s with a MS73 probe (Sonopuls HD70, Bandelin) at an amplitude of 42 μm and a power of < 10 W. Cells were filtered on polycarbonate membrane filters (Isopore, GTTP, 0.2 μm pore size, Millipore). In situ hybridisations with horseradish peroxidase (HRP)‐labelled probes followed by fluorescently‐labelled tyramine signal amplification (catalysed reporter deposition; CARD) were carried out as described previously (Pernthaler et al. 2002; Kleindienst et al. 2012). Tyramides were labelled with Alexa488. Permeabilisation was done by incubating the filter sections in 10 mg mL^−1^ lysozyme for 30–60 min at 37°C. For archaea and SCA2 cells, an additional permeabilisation step was required following the lysozyme treatment: filters were further incubated with 15 μg mL^−1^ proteinase K in TE (50 mM EDTA, 100 mM Tris) buffer for 3 min at room temperature. Inactivation of endogenous peroxidases was done by incubating the filters in 0.15% H_2_O_2_ in methanol for 30 min at room temperature. Probes were purchased from biomers.net (Ulm, Germany). Formamide concentrations required for specific hybridisation are given in Table S1.

### 2.7 Biovolume Calculation

Biovolumes of target cells present in the incubations and in the environmental samples were calculated based on their cell sizes measured after CARD‐FISH. Cell length and width were determined in images obtained with an epifluorescence microscope (Nikon eclipse 50i, Düsseldorf, Germany) equipped with an AxioCam MRc camera (Carl Zeiss, Jena, Germany). From the incubations, a total of 25 SCA1, 71 SCA2 and 24 LCA2 cells were measured. From the environmental samples, we aimed to measure at least 10 cells per sample and clade. For some samples, however, we could not obtain 10 images of sufficient quality and fewer cells were used for calculation. Biovolume for rod‐shaped cells was calculated according to π·D^2^·(L/4‐D/12), where D and L are the cell diameter and length, respectively. For coccoid cells, biovolume was calculated according to 4/3·π·r^3^, where r is the cell radius.

### 2.8 Nanometre Scale Secondary Ion Mass Spectrometry

Sonication was followed by density gradient centrifugation to enrich the detached cells for nanometre scale secondary ion mass spectroscopy (NanoSIMS). 200 μL of the sample was mixed with 800 μL 1× PBS. One millilitre Histodenz nonionic density gradient medium (Sigma Aldrich, Taufkirchen, Germany) (60% w/v in 1× PBS) was carefully added with a syringe and needle underneath the sample. Centrifugation was performed at 14,000 × g for 20 min at 20°C. After the centrifugation, 1500 μL of the supernatant was sampled, while the sediment pellet was re‐used for an additional density gradient centrifugation step. The combined supernatants were filtered on three polycarbonate membrane filters (Isopore, GTTP, 5 mm diameter, 0.2 μm pore size; Millipore) pre‐sputtered with a 10 nm thick gold/palladium (Au/Pd) layer (sputter coater; GaLa—Gabler Labour Instrumente; Germany).

Selection of time points for sampling the incubations for NanoSIMS analysis was based on the measured production of sulphide (ca. 2 mM sulphide in all incubations; and in addition ca. 4 mM sulphide for the second NanoSIMS time point of the Amon MV‐butane incubation), cell counts, and ^13^C enrichments of the bulk carbon pools, that is, TOC and DIC. CARD‐FISH signals were used to identify SRB of the target groups SCA1, SCA2, and LCA2. Fields on the filter containing target cells were marked using laser micro‐dissection (LMD model DM6000B; Leica Microsystems).

Fields on the Au/Pd‐coated polycarbonate filters containing hybridised target cells were analysed with a NanoSIMS 50 L (Cameca, Gennevilliers Cedex‐France) at the Max Planck Institute for Marine Microbiology in Bremen, Germany. First, areas of interest were pre‐sputtered with a primary Cs^+^ ion beam of 100 pA to remove surface contamination and to implant Cs^+^ ions in the sample until an approximately stable ion emission yield was achieved. Subsequently, the primary Cs^+^ beam (current between 0.8 and 1 pA, beam diameter between 50 and 100 nm) was rastered across the sample area (10 × 10 μm to 20 × 20 μm in size, 256 × 256 pixels resolution) with a dwell time of 1 ms per pixel while the counts of the emitted secondary ions ^12^C^−^, ^13^C^−^, ^19^F^−^, ^12^C^14^N^−^ and ^32^S^−^ were simultaneously recorded by separate electron multiplier detectors. To minimise interferences for ^13^C^−^, the instrument was tuned for high mass resolution (around 7000 MRP).

NanoSIMS data were processed with the Look@NanoSIMS program (Polerecky et al. 2012). First, individual planes of detected secondary ions were drift‐corrected based on the ^12^C^14^N^−^ ion counts and accumulated. Subsequently, CARD‐FISH images of the same field of view (FOV) were aligned and overlaid with the accumulated ^12^C^14^N^−^ images and used to draw regions of interest (ROIs) corresponding to individual target cells. Finally, the ^13^C atom fraction in the target cells was calculated as x(^13^C) = ^13^C^−^/(^12^C^−^ + ^13^C^−^), where ^13^C^−^ and ^12^C^−^ are total counts of the respective secondary ions accumulated over all pixels in the corresponding ROIs. The depth profiles of the ^13^C atom fractions did not exhibit significant trends with depth (data not shown), which justified this calculation (Polerecky et al. 2012).

All ^13^C enrichment data, including those for the target cells and for the bulk DIC and TOC pools, are presented as excess ^13^C atom fractions, calculated as the difference between the atom fraction at time point *t* and the corresponding initial value, that is, 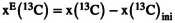.

### 2.9 Modelling of Carbon Flows During the Incubation Experiment

A modelling approach was used to estimate the rates of cellular activity of the target cells and evaluate their impact on the carbon and sulphur fluxes within the studied system. The model integrates present observations with assumptions informed from previous studies, as outlined below. A detailed description of the model is available in the Supporting Information.

#### 2.9.1 Assumptions

The model is based on several assumptions. First, we assumed that the number of cells in the target population, *N*, increased during the incubation according to the differential equation d*N*/d*t* = *k* × *N* × *f*_lim_, where *k* is the rate constant and *f*_lim_ is a factor describing growth limitation (*f*_lim_ ≤ 1). In general, *f*_lim_ is a product of factors that account for growth limitation by substrate availability (e.g., Michaelis–Menten kinetics) and for the lag and stationary phases in the growth of bacterial populations, as proposed by Baranyi et al. (1993) and Baranyi and Roberts (1994). In the present study, it was sufficient to use *f*_lim_ in the form of *f*_lim_ = (1 − *N*/*N*_max_), which accounts for the stationary growth phase, while implementing substrate limitation and lag‐phase was not necessary. Thus, the growth of the target populations was adequately described by the classical logistic growth model.

Second, we assumed that the carbon content of the target cell population, *C*, is related to the cell counts according to *C* = *N* × *C*_cell_, where *C*_cell_ is the carbon content of an average individual target cell. The cellular carbon content was calculated from the cellular biovolume, determined through microscopy, and a biovolume‐normalised carbon content of 55 fg C μm^−3^, as determined for the closely‐related *Desulfosarcina* sp. strain BuS5 (Jaekel et al. 2013).

Third, we assumed that the carbon assimilated by the target cells originated from two carbon pools: part *β* originated from the added ^13^C‐labelled alkane, while the remaining part 1 − *β* originated from the DIC pool. Based on previous observations of carbon assimilation in sulphate‐reducing bacteria, we considered the value of *β* = 0.7 (Widdel and Hansen 1992; Rabus et al. 2006).

Fourth, we assumed that part *α* of the added alkane was assimilated, directly leading to an increasing ^13^C‐enrichment of the target cells, while the remaining part 1 − *α* was used for energy generation, leading to the production of ^13^C‐labelled DIC (and thus indirectly leading to an increasing ^13^C‐enrichment of the target cells) and hydrogen sulphide (H_2_S). In this step, we additionally assumed that the produced ^13^C‐DIC was fully mixed with the bulk DIC pool, that is, there was no mass transfer limitation that could lead to a preferential assimilation of the ^13^C‐DIC newly produced from the oxidation of the ^13^C‐labelled alkane. Based on previous studies with hydrocarbon‐degrading sulphate‐reducing bacteria, we considered the value of *α* = 0.1, that is, 10% of the hydrocarbon substrate being assimilated into cell mass (Rabus et al. 2006; Widdel and Musat 2010).

Fifth, the DIC and H_2_S production were assumed to follow the stoichiometry of the complete oxidation of butane and dodecane by sulphate‐reducing bacteria, that is,

$$\left(\text{butane oxidation}\right)\;C_{4}H_{10}+3.25{\mathrm{SO}}_{4}^{2-}+2.5H^{+}\to 4{\mathrm{HCO}}_{3}^{-}+3.25H_{2}S+H_{2}O$$

$$\begin{array}{c} \left(\text{dodecane oxidation}\right)\;C_{12}H_{26}+9.25{\mathrm{SO}}_{4}^{2-}+6.5H^{+}\\ \;\to 12{\mathrm{HCO}}_{3}^{-}+9.25H_{2}S+H_{2}O \end{array}$$

Finally, we assumed that due to the dilution of the intracellular carbon pool associated with the application of the CARD‐FISH procedure, the ^13^C enrichment of the target cells determined by the NanoSIMS analysis was by a factor *f*_dil_ lower than the true ^13^C enrichment resulting from the assimilation of the labelled substrates (^13^C‐alkane and ^13^C‐DIC) during the incubation. To date, the dilution of the intracellular ^13^C pool due to CARD‐FISH was determined for a limited number of strains (Musat et al. 2014, 2016). Here, we assumed a dilution factor of *f*_dil_ = 0.57, as previously determined for *Pseudomonas putida* (Musat et al. 2014), based on the similarity in cell structure and size (Gram negative, rods) to the SCA and LCA groups.

#### 2.9.2 Fitting of the Experimental Data

Based on the assumptions, we formulated mathematical equations that describe the flow of carbon isotopes ^12^C and ^13^C between the relevant carbon pools during the incubation, including alkane, target cell biomass, total organic carbon (TOC), and DIC (see Supporting Information). These equations were solved numerically in R (R Core Team 2021) using the R‐package deSolve (Soetaert et al. 2010) and were used for fitting the experimental data.

The fitting proceeded in two steps. First, we used the target cell counts combined with their cellular ^13^C enrichments determined by NanoSIMS to constrain the rate constant *k*, the initial cell count *N*_0_, and the maximal cell count *N*_max_. In the second step, these values were then used to predict the removal of the added alkane and the accompanying increase in the ^13^C enrichment of the TOC and DIC pools. Comparison with the experimental data revealed that the model underestimated both alkane removal and ^13^C‐DIC and ^13^C‐TOC production, suggesting that the active biomass inferred from the measured cell counts and cellular biovolumes was insufficient to explain the experimental data. To estimate this “undetected biomass”, we varied the initial (*N*_0_) and maximal (*N*_max_) cell counts obtained in the first step by a factor hereafter referred to as the biomass increase factor, BIF, until the model matched the measured removal of alkane and production of ^13^C‐DIC and ^13^C‐TOC. In this second step, no variation in the rate constant *k* was necessary to improve the fit of the data with the model. Therefore, this critical model parameter is essentially constrained by the NanoSIMS data and the assumed value of the dilution factor *f*_dil_ = 0.57, whereas BIF is constrained by the concentration and isotopic composition of the bulk carbon pools (alkane, DIC and TOC).

#### 2.9.3 Calculation of the Cellular Rates

In the final step, we used the estimated rate constant *k* (in d^−1^), the average cellular carbon content *C*_cell_ (in mol C cell^−1^), and formulas in Equations ((1), (2), (3), (4)) to calculate the rates characterising the activity of the target cells, including the cellular rate of carbon assimilation (*r*_*C*_, in mol C d^−1^ cell^−1^), alkane removal (*r*_alkane_, in mol alkane d^−1^ cell^−1^), DIC production (*r*_DIC_, in mol C d^−1^ cell^−1^), and sulphate reduction (*r*_SR_, in mol S d^−1^ cell^−1^).

(1) $$r_{C}=k\cdot C_{\text{cell}}$$

(2) $$r_{\text{alkane}}=\frac{\beta }{\alpha \cdot \nu_{\text{alkane}}}\cdot r_{C}$$

(3) $$r_{\mathrm{DIC}}=\left[\frac{\beta }{\alpha }-1\right]\cdot r_{C}$$

(4) $$r_{\mathrm{SR}}=\frac{1-\alpha }{\alpha }\cdot \beta \cdot \frac{\nu_{H_{2}S}}{\nu_{\text{alkane}}}\cdot r_{C}$$

In these formulas, the stoichiometric coefficient *ν*_alkane_ is equal to 4 for butane and 12 for dodecane, and $\nu_{H_{2}S}=\left(3\cdot \nu_{\text{alkane}}+1\right)/4$. As explained in the Supporting Information, the values obtained by Equations ((1), (2), (3), (4)) represent maximal rates of cellular activity, that is, rates at which the cells would convert substrate into biomass, DIC, and H_2_S if they were growing exponentially (i.e., no substrate‐limitation, far from the lag and stationary growth phases).

### 2.10 Extrapolation of Rates for Specific Groups

To investigate the potential contribution of the target groups to sulphate reduction, the in situ abundance and biovolumes of SCA1, SCA2, and LCA2 cells were determined by CARD‐FISH. Then, cellular rates were calculated according to Equations ((1), (2), (3), (4)) using the mean cell biovolumes and the corresponding rate constants estimated from the incubation experiments. Finally, the cellular rates were multiplied by cell abundance to estimate the sediment‐specific sulphate reduction rate for the target groups.

## 3 Results

### 3.1 Consumption of ^13^C‐Labelled Butane and Dodecane During Incubation Experiments

**FIGURE 1:**
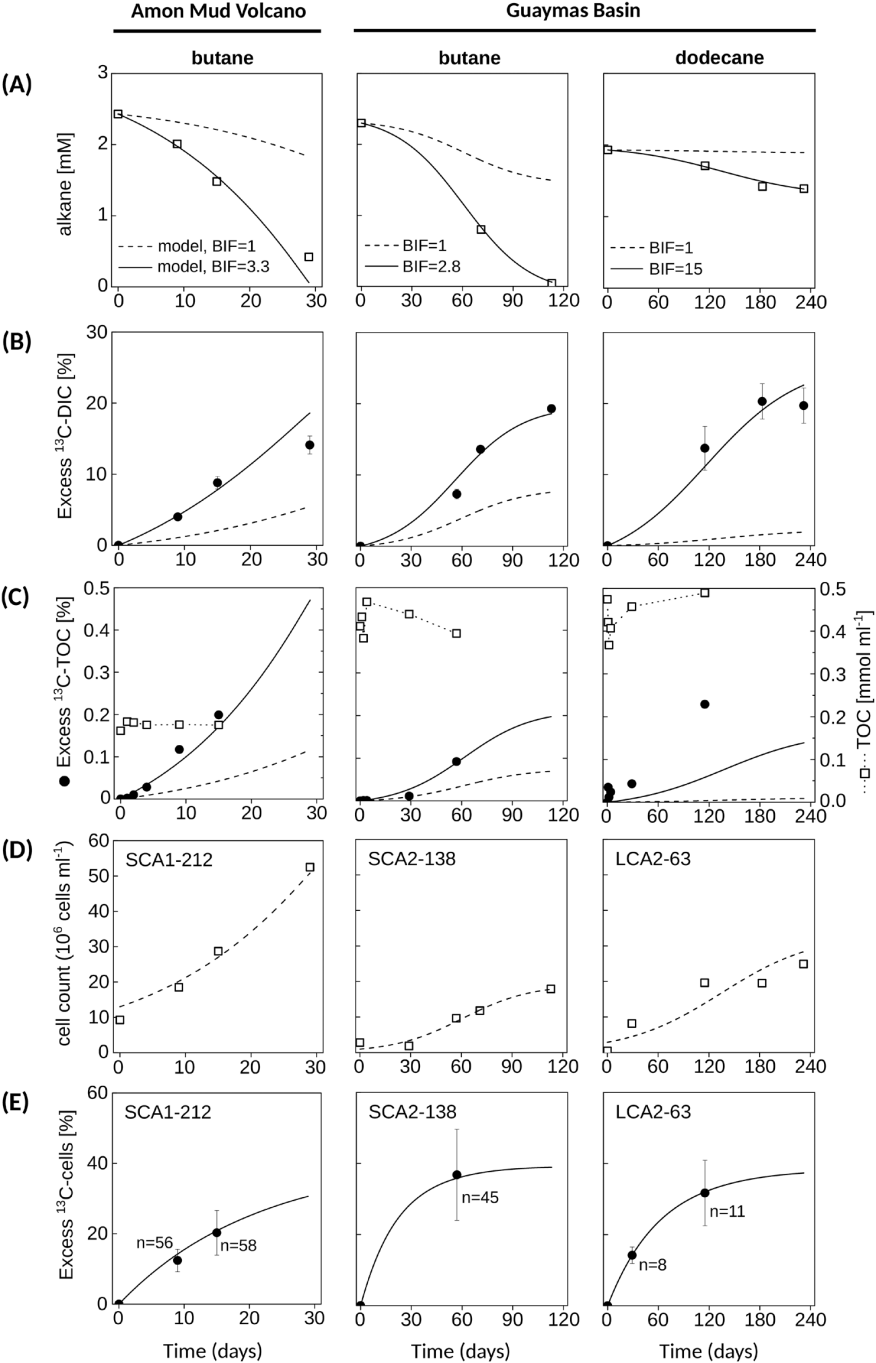
Characteristics of the relevant carbon pools in sediment slurries incubated with added ^13^C‐alkane (butane or dodecane). The shown characteristics include (A) porewater alkane concentrations, (B) ^13^C enrichment of the porewater DIC, (C) ^13^C enrichment (left axis, filled symbols) and concentration (right axis, open symbols) of the bulk TOC, (D) cell counts of the target cells, and (E) ^13^C enrichment of the target cells (data point is the mean value, error‐bar corresponds to 1 × SD of *n* individual cells). The ^13^C enrichment data are presented as excess ^13^C atom fractions in atom %. In all panels, points show experimental data while lines show modelled data. The modelled data assumed that the biomass of the alkane degrading population was the same as derived from the measured abundance and biovolume of the target cells (dashed line), or was increased by a factor BIF (solid line). Alkane concentrations (A), DIC ^13^C enrichment (B) and cell abundance data (D) were taken from Kleindienst et al. (2014).

Sediment slurries were amended with either ^13^C‐labelled butane or ^13^C‐labelled dodecane and incubated with sulphate as the terminal electron acceptor under anoxic conditions. Butane was consumed within 29 days and 113 days in the Amon MV‐butane and Guaymas Basin‐butane incubations, respectively (Figure 1A). In both cases, butane consumption was accompanied by a strong increase in excess ^13^C‐DIC (Figure 1B), indicating complete butane oxidation to CO_2_. Dodecane was consumed much more slowly than butane, with less than 40% of the provided dodecane degraded over the 232 days incubation period (Figure 1A). Similar to the butane incubations, consumption of ^13^C‐dodecane was accompanied by an increase in excess ^13^C‐DIC, indicating active microbial degradation of the substrate (Figure 1B). Although similar levels of ^13^C‐DIC enrichments were observed in both the butane and dodecane assays, the dodecane incubations required more time to reach ^13^C‐DIC enrichments comparable to those in the butane incubations. For example, a ^13^C‐DIC enrichment of 15% was detected after about 30–70 days in the butane incubations, while the Guaymas Basin incubations with dodecane took 115 days to reach a comparable enrichment (Figure 1B). This was likely due to poor substrate bioavailability, owing to the low solubility of dodecane in water. In assays for both substrates, the ^13^C enrichment of the total organic carbon (TOC) pool also increased over time (Figure 1C), suggesting that the added alkanes were utilised as a carbon source for growth.

### 3.2 Growth of SCA and LCA Cells During Incubation Experiments

**FIGURE 2:**
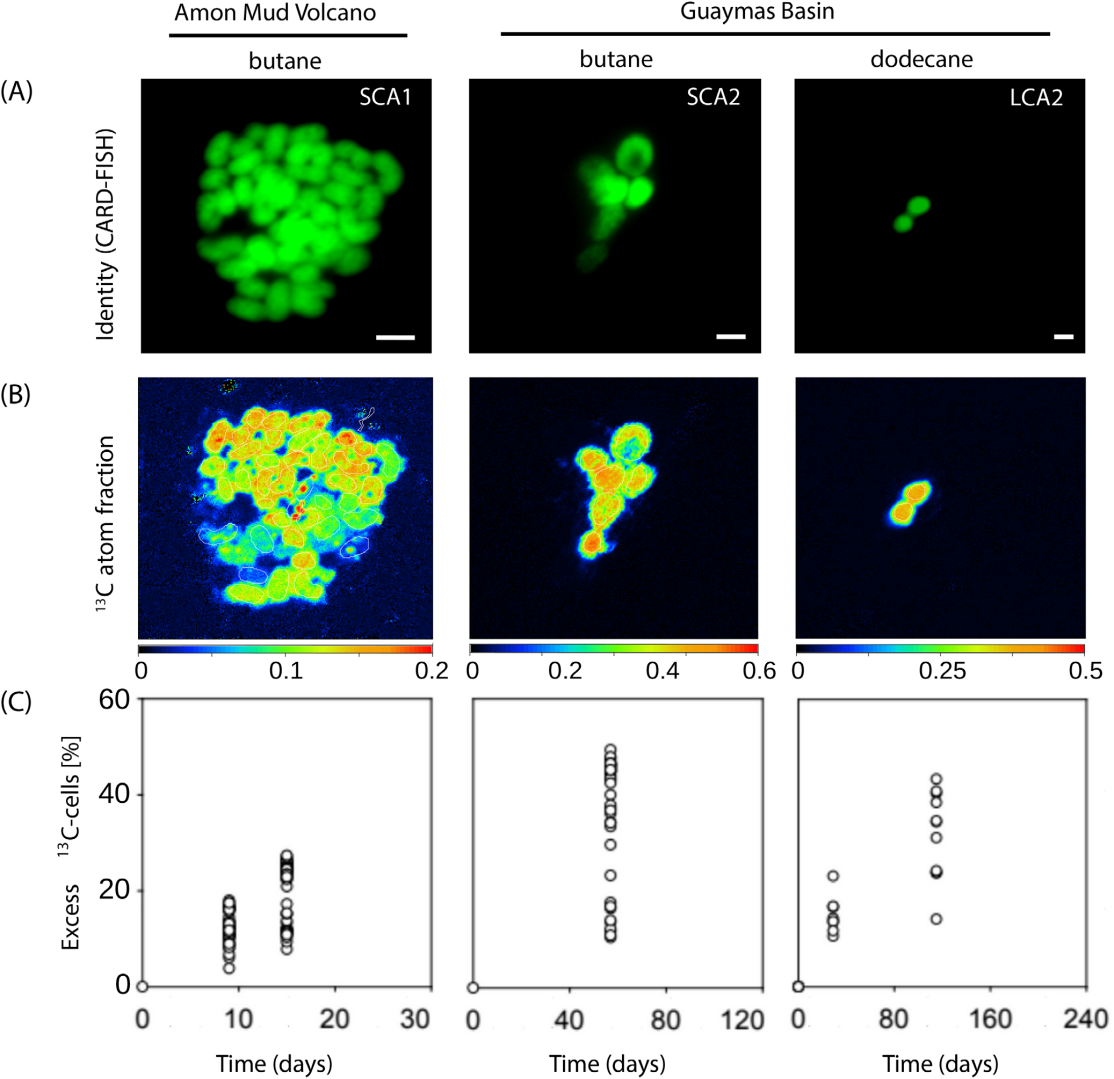
Correlative imaging of target cells and their alkane‐derived carbon assimilation activity. (A) CARD‐FISH images. (B) Corresponding NanoSIMS images of ^13^C atom fraction. (C) Summary of excess ^13^C atom fractions in all target cells measured by NanoSIMS in this study. Shown are data for specific butane‐ or dodecane‐degrading cells from Amon MV and Guaymas Basin seep sediments identified by specific probes for SCA1, SCA2, and LCA2. In panel B, note differences in colour scales among the sites. Scale bars represent 2 μm.

Target clades of sulphate‐reducing bacteria were identified and quantified using CARD‐FISH with clade‐specific probes: SCA1 in the Amon MV‐butane incubations, SCA2 in the Guaymas Basin‐butane incubations, and LCA2 in the Guaymas Basin dodecane incubations. Along with the increase of ^13^C‐TOC, the absolute and relative abundances of SCA1, SCA2, and LCA2 increased during the incubations (Figure 1D, Tables S2 and S3). Specifically, the cell abundances increased 6‐fold for SCA1 and SCA2 and 57‐fold for LCA2 in the course of the experiment (Table S2). The SCA1 cells in the Amon MV‐butane incubations occurred mainly loosely aggregated (Figure 2A). Both cell numbers within aggregates and overall aggregate sizes (up to 12 μm in diameter) increased over the course of incubation. Cell morphology was oval or slightly curved, and although individual cell biovolumes varied (1.66 ± 0.42 μm^3^; coefficient of variation CV = 26%; Table S3), the average biovolume remained constant over the incubation period. The SCA2 cells in the Guaymas Basin‐butane incubations showed a similar aggregation behaviour. Their length increased during the incubation (ranged between 1.3 to 4 μm). Their biovolume was on average about 3.2‐fold larger than that of the SCA1 cells and considerably variable among cells (5.33 ± 3.54 μm^3^; CV = 66%; Table S3). The LCA2 cells in the Guaymas Basin‐dodecane incubation were coccoid or slightly oval and did not aggregate (Figure 2). Their diameter (~1 μm) and biovolume (0.48 ± 0.17 μm^3^; CV = 35%; Table S3) were constant during the incubation. Assuming a biovolume‐normalised carbon content of 55 fg μm^−3^, as determined for the closest cultivated representative *Desulfosarcina aeriophaga* BuS5 (Jaekel et al. 2013), the estimated average cellular carbon contents were 7.6 fmol cell^−1^ for SCA1, 24.4 fmol cell^−1^ for SCA2, and 2.2 fmol cell^−1^ for LCA2 cells (Table S3).

### 3.3 Assimilation of ^13^C‐Labelled Alkanes by SCA and LCA Cells During Incubation Experiments

To determine if cells of the SCA and LCA clades were involved in alkane oxidation, subsamples of the assays with ^13^C‐alkanes were analysed by NanoSIMS‐based chemical imaging with a single‐cell resolution. The underlying hypothesis was that alkane‐oxidising SRB, which are known to assimilate alkane‐derived carbon into biomass, will become progressively enriched in ^13^C. The incubation times with labelled alkanes were kept as short as possible to minimise the transfer of labelled metabolites from primary alkane degraders to secondary consumers.

**FIGURE 3:**
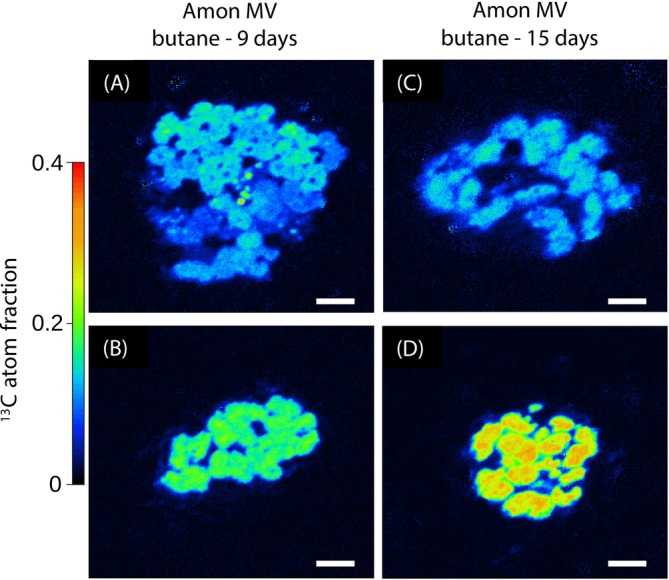
NanoSIMS images of selected aggregate‐forming butane‐degrading target cells (group SCA1) from Amon Mud Volcano marine seep sediments. Shown are ^13^C atom fraction images of cells incubated with ^13^C‐butane for 9 days (A, B) and 15 days (C, D). The apparent enrichment in ^13^C was similar among cells within one aggregate, whereas it differed between aggregates, suggesting at least two subpopulations with distinct levels of substrate assimilation. Scale bar indicates 2 μm.

For the three target groups, we analysed between 19 and 114 individual cells by NanoSIMS. All target cells showed a strong increase in their ^13^C enrichment over time (Figure 1E). At any given time point, the cellular ^13^C enrichment was greater than that of the DIC pool, indicating the assimilation of the ^13^C‐labelled alkane into cell mass. The ^13^C enrichment was variable among individual cells (Figure 2B,C). The enrichment in ^13^C, quantified as excess ^13^C atom fraction, reached 20% ± 6% (standard deviation; SD) after 15 days in the SCA1 cells, 37% ± 13% (SD) after 57 days in the SCA2 cells, and 32% ± 9% (SD) after 115 days in the LCA2 cells. The ^13^C enrichment values were similar for cells within the same aggregate but differed among aggregates, an observation most prominent for SCA1 cells in the Amon MV‐butane incubation (Figure 3).

### 3.4 Estimating the Cellular Activities of SCA and LCA Cells During Incubation Experiments

The rate constants for the target cells were estimated by fitting the cell counts (determined by combining epifluorescence microscopy with CARD‐FISH; Figure 1D) and the cellular ^13^C enrichment data (measured by NanoSIMS; Figure 1E) with the model (see Experimental Procedures for details). The rate constant *k* was similar for the butane‐degrading cells SCA1 (0.055 d^−1^) and SCA2 (0.049 d^−1^), whereas it was about three‐fold lower for the dodecane‐degrading cells LCA2 (0.018 d^−1^) (Table 1). These rate constants translate to doubling times of about 13 days (SCA1), 14 days (SCA2) and 39 days (LCA2) (Table 1).

**TABLE 1:** Estimated rates of activity of the target cells. All values correspond to the situation where the cellular activity is not limited by the availability of substrate or other, non‐specific limitation factors.

Incubation (target group)	*k*, rate constant^a^ (d^−1^)	*τ*, doubling time^b^ (d)	*N*_ini_, Initial cell count [10^6^ cells mL^−1^)	C_cell_, cellular carbon content (fmol C cell^−1^)	Cell‐specific rates^c^				α^e^	β^f^	dil^g^	BIF^h^	Rel. SE^i^ (%)
					Carbon assimil.^d^ (fmol C cell^−1^ d^−1^)	Alkane removal (fmol alkane cell^−1^ d^−1^)	Net DIC production (fmol C cell^−1^ d^−1^)	Sulphate reduction (fmol S cell^−1^ d^−1^)					
Amon MV‐Butane (SCA1)	0.055	12.6	13	7.6	0.42	0.73	2.51	2.14	0.1	0.7	0.57	3.3	9
Guaymas Basin‐butane (SCA2)	0.049	14.1	0.97	24.4	1.2	2.11	7.24	6.18	0.1	0.7	0.57	2.8	13
Guaymas Basin‐dodecane (LCA2)	0.018	38.5	2.9	2.2	0.04	0.023	0.24	0.19	0.1	0.7	0.57	15	16

^a^ Value constrained by NanoSIMS data in the present study.

^b^ Calculated according to *τ* = ln(2)/*k*.

^c^ Values derived from the rate constant, cellular carbon content, and parameters *α* and *β*, as described in Experimental Procedures.

^d^ To convert the rate of alkane‐derived carbon assimilation to the rate of alkane assimilation (fmol alkane cell^−1^ d^−1^), divide the value by 4 (for butane) and 12 (for dodecane).

^e^ Parameter *α* describes the fraction of alkane assimilated into biomass. Fraction 1 − *α* is used for energy generation. Value assumed based on literature (Rabus et al. 2006; Widdel and Musat 2010).

^f^ Parameter *β* describes the fraction of assimilated C originating from alkane. The rest of the assimilated C (fraction 1 − *β*) originates from DIC. Value assumed based on literature (Widdel and Hansen 1992; Rabus et al. 2006).

^g^ Label dilution factor due to CARD‐FISH: ratio between the cell‐specific ^13^C/C ratio measured by NanoSIMS after the application of CARD‐FISH and the corresponding value not affected by CARD‐FISH. Value assumed based on literature (Musat et al. 2014).

^h^ Biomass increase factor (BIF): A factor by which the initial biomass of the target cell population would need to be increased to match the observed bulk rates of alkane removal and ^13^C‐DIC and ^13^C‐TOC accumulation during the incubation experiments. Value constrained by combining the bulk and single‐cell measurements, as described in Experimental Procedures.

^i^ Relative standard error (Rel. SE) represents the uncertainty of the estimated rates. Estimated based on two contributions: variability of the cellular ^13^C‐enrichment measured by NanoSIMS, and variability of the cell's biovolume (and thus of the cell's carbon content) measured by fluorescence microscopy.

By considering the average cellular carbon content estimated from microscopy‐derived cellular biovolumes (Table S3), the rate constant of 0.055 d^−1^ for the SCA1 cells translates to the cellular carbon assimilation rate of 0.42 fmol C cell^−1^ d^−1^ (1 fmol = 10^−15^ mol), alkane removal rate of 0.73 fmol C_4_ cell^−1^ d^−1^, DIC production rate of 2.51 fmol C cell^−1^ d^−1^, and sulphate reduction rate of 2.14 fmol S cell^−1^ d^−1^ (Table 1). The cellular rates were about three‐fold larger for the SCA2 cells (mostly due to their roughly 3‐fold larger biovolume, Table S3), and about 10‐fold lower for the LCA2 cells (including the dodecane removal rate, when expressed in the units of C rather than dodecane) (Table 1). By combining the uncertainties in the determination of the mean cellular biovolume (Table S3) and of the cellular ^13^C enrichment (Figure 1E), the relative standard error of the estimated cellular rates ranged between 9% and 16% (Table 1, column Rel. SE).

By using the cell counts, cellular biovolumes and cellular ^13^C enrichments of the target cells as the primary data to constrain the model, the bulk rates of alkane removal and excess ^13^C‐DIC and ^13^C‐TOC production predicted by the model underestimated the corresponding rates measured during our incubations (compare symbols with dashed lines in Figure 1A–C). The minimum requirement to overcome this discrepancy was to assume that the total biomass of the alkane‐degrading community was greater than the estimated biomass of the target groups. Since the latter was calculated using the cell counts, biovolumes and carbon density, this increase could be achieved by proportionally increasing any of these three parameters or a combination of the three. To match the measured data, the factor by which the active biomass needed to be increased, while keeping all the other model parameters unchanged, was determined to be 3.3 and 2.8 for the ^13^C‐butane incubations in the Amon MV and Guaymas Basin sediments, respectively, and 15 for the ^13^C‐dodecane incubation in the Guaymas Basin sediment (Table 1, column “biomass increase factor”, BIF). For the ^13^C‐butane incubations (both Amon MV and Guaymas Basin) this biomass adjustment resulted in a good agreement between the model predictions and all observed data, whereas for the ^13^C‐dodecane incubation in Guaymas Basin the bulk ^13^C‐TOC production was still underestimated by the model (compare solid lines with symbols in Figure 1A–C).

### 3.5 Growth of Other Sulfate Reducers During Incubation Experiments

To investigate the “undetected” fraction of the alkane‐degrading community in our experimental slurries, we quantified members of the re‐classified former *Deltaproteobacteria*, specifically the *Desulfosarcina/Desulfococcus* (DSS) branch, as well as *Archaea*, using specific FISH probes (Table S1). According to the SILVA database 138.1_SSURef_NR99 (release July 2024; Quast et al. 2013; Yilmaz et al. 2014), probe set Delta495a‐c (Loy et al. 2002) covers 87% of *Desulfobacterota*, 73% of *Bdellovibrionota*, and 23% of *Myxococcota*, as well as several other clades without a taxonomic name (e.g., Sva0485, NB1‐j). At time point zero, archaeal cell numbers in our Guaymas Basin incubations were about 6‐fold higher than those of cells detected by the probe set Delta495a‐c (Table S2). Among the archaea, we detected *Ca*. Syntrophoarchaeum (probe SYNA407; Laso‐Pérez et al. 2016), *Ca*. Argoarchaeum, and *Ca*. Ethanoperedens (probe GoM‐ArcI‐660; Hahn et al. 2020). Quantification of these groups, however, was impossible due to their growth in few but large cell aggregates.

In the Amon MV‐butane incubations, the number of non‐SCA1 DSS cells increased by 5.4 × 10^7^ cells mL^−1^ over the course of the experiment, based on the difference between cell counts detected with probes DSS658 and SCA1‐212 (Table S2), indicating the growth of other potential butane degraders. By contrast, in the Guaymas Basin‐dodecane incubation, LCA2 cell counts increased to a similar extent as total DSS cell counts (Table S2), indicating that likely no other dodecane‐degrading SRB of the DSS clade besides LCA2 were enriched. However, other non‐DSS bacteria of the former class of *Deltaproteobacteria* increased in cell numbers by a factor of 12, which is twice the increase observed for DSS cells, indicating that, in addition to our target groups, other alkane degraders outside the DSS clade and *Archaea* were likely active in the Guaymas Basin sediments.

### 3.6 Potential Impact of SCA and LCA Clades in Hydrocarbon Seep Sediments

To assess the potential contributions of SCA and LCA clades to geochemical cycles, their abundance was quantified in representative, globally distributed hydrocarbon seeps (Table 2). At most investigated sites, SCA1 comprised less than 1% of total cell counts. Higher abundances of up to 3% were found in sediments from the Chapopote asphalt volcano in the southern Gulf of Mexico (site GeoB106196; 1.6 × 10^8^ cells mL^−1^), at seeps in the Tommeliten area (site 1274 K3; 1.4 × 10^8^ cells mL^−1^), and at Amon MV (site 825; 2.0 × 10^7^ cells mL^−1^). SCA1 cells were rod‐shaped or slightly curved, resembling the morphology of previously described members of the SCA1 group (i.e., strain BuS5 and Butane‐GMe12) (Kniemeyer et al. 2007; Jaekel et al. 2013). They were found as aggregated or non‐aggregated cells. The relative abundance of the SCA2 clade was below 1% of total cell counts at all sites, except at the Tommeliten site 1274‐K3, where it accounted for 1.4%. SCA2 were coccoid to rod‐shaped, non‐aggregated cells. Highest abundances of LCA2 (~0.8% of total cells, 1.9 × 10^8^ cells mL^−1^) were detected within a surface mat on brittle asphalt at the Chapopote asphalt volcano (site GeoB10619‐13; Table 2). Despite low abundances with respect to the total cell numbers, members of SCA1, SCA2, and LCA2 clades had a major contribution to the sulphate‐reducing microbial communities, accounting for up to 31%, 9%, and 6% of all cells detected by probe Delta495a‐c, respectively (Table 2).

**TABLE 2:** Distribution and in situ abundance of the re‐classified class *Deltaproteobacteria*, *Desulfosarcina/Desulfococcus* branch of *Desulfobacterota* (DSS), and alkane‐degrading clades SCA1, SCA2 and LCA2 as detected by CARD‐FISH at diverse marine seeps. Probes used: Delta495a‐c targeting *Deltaproteobacteria;* probe DSS658 targeting DSS; SCA1‐212ab targeting SCA1; SCA2‐138 targeting SCA2; and LCA2‐63 targeting LCA2.

Habitat	Station	Sample type	Sediment depth (cm)	*Delta‐proteobacteria* ^a^		DSS^a^		SCA1	SCA2	LCA2
				Free‐living cells (10^8^ cm^−3^)	Total cells^b^	Free‐living cells	Total cells^b^	Total cells (10^6^ cm^3^)	Total cells	Total cells
Northern Gulf of Mexico	GoM156	Gassy, near oily sediment	3	2.3	11.6	1.6	9.8	5.2	5.2	5.2
	GoM161	Oily sediment, hydrate	5	3.2	3.2	2.5	2.5	6.4	6.4	6.4
Southern GoM Chapopote Asphalt Volcano	GoM140	Oily sediment, hydrate	1	0.3	NA	0.3	0.3	2.1	2.1	ND
	GeoB10619‐13	Mat on brittle asphalt		NA	NA	11.1	NA	62.3	62.3	186.9
	GeoB10619‐6	Oily sediments	1.25	6.2	NA	3.7	NA	160.4	14.1	14.1
	GeoB10625‐16	Asphalt with white precipitate		NA	NA	0.7	NA	4.7	ND	ND
	GeoB10625‐9	Oily sediments	3.75	4.9	NA	2.5	NA	9.7	9.7	9.7
			13.75	4.2	NA	2	NA	18.3	18.3	6.1
Guaymas Basin	GB4489‐1	Hydrocarbon sediments	0.5	19.2	19.2	2.9	2.9	ND	44.0^c^	44.0^c^
			2.5	3.2	3.2	1	1	5.0^c^	5.0^c^	ND
Haakon Mosby Mud Volcano	ATL19	Sediment below Beggiatoa mat	1.5	7.9	7.9	2.3	2.3	ND	ND	12
			8.5	0.2	0.2	0.1	0.1	ND	ND	ND
	ATL22	Sediment below Pogonophora field	3.5	5.1	5.1	3.6	3.6	ND	ND	ND
Amon Mud Volcano	Amon MV760	Sediment below Beggiatoa mat	2.5	0.8	6	0.3	5	4,8^c^	1.6^c^	4.8^c^
	Amon MV825	Sediment below bacterial mat	0.5	1.7	11.6	1.4	11.3	5.1^c^	5.1^c^	ND
			4.5	0.6	4.8	0.6	2.6	20.2^c^	ND	1.6^c^
Hydrate Ridge	HR19	Sediment below Beggiatoa mat	4.5	3.9	162.5	2.9	153.4	19	ND	ND
	HR38	Sediment below Calyptogena field	12.5	0.4	68.2	0.6	67.2	3.3	3.3	ND
Tommeliten	1274‐K1	Methane seep sediment	1.5	3	3.2	2.8	2.8	ND	ND	ND
	1274‐K2	Methane seep sediment	1.5	7.3	7.8	3	3	37.5	12.5	12.5
	1274‐K3	Methane seep sediment	1.5	10.1	10.9	2	2	142.5	85.3	ND
			5.5	4.6	5.6	1.5	1.5	26.3	37.9	ND
			8	6.4	7	1.6	1.6	ND	8.7	ND

Abbreviations: NA, sample not analysed; ND, sample analysed but cells not detected.

^a^ Numbers taken from Kleindienst et al. (2012).

^b^ Numbers include bacteria living in aggregates with archaeal partners.

^c^ Numbers taken from Kleindienst et al. (2014).

In situ cell biovolumes were determined based on CARD‐FISH signals. They varied among sites and ranged between 0.17 and 1.33 μm^3^ for SCA1 (mean: 0.67, SD: 0.43 μm^3^), 0.17–0.96 μm^3^ for SCA2 (mean: 0.44, SD: 0.41 μm^3^), and 0.10–0.56 μm^3^ for LCA2 (mean: 0.32, SD: 0.19 μm^3^) (Table S4). Subsequently, the biovolume‐normalised carbon content of 55 fg C μm^−3^ (Jaekel et al. 2013; Stryhanyuk et al. 2018) was used to calculate clade‐specific sulphate reduction rates using the rate constants constrained from the incubation experiments (Table 1). Generally, SCA1 cells had the greatest contribution to sulphate reduction: their extrapolated sulphate‐reduction rates ranged between 0.7 and 163.0 nmol cm^−3^ sediment d^−1^ with the highest rates at the Asphalt Volcano (site GeoB10619‐6) (Table 3). Extrapolated sulphate reduction rates for SCA2 cells ranged between 0.9 and 39.7 nmol cm^−3^ d^−1^ (Table 3). Sites with high extrapolated sulphate‐reduction rates were Guaymas Basin (site 4489–1) with 25.9 nmol cm^−3^ d^−1^, the Asphalt Volcano (site GeoB10619‐13) with 27.9 nmol cm^−3^ d^−1^, and Tommeliten (site 1274‐K3) with 39.7 nmol cm^−3^ d^−1^. Extrapolated sulphate‐reduction rates for LCA2 ranged from 0.2 to 7.6 nmol cm^−3^ d^−1^, being highest at the Asphalt Volcano (site GeoB10619‐13) (Table 3).

**TABLE 3:** Extrapolation of cellular rates (sulphate reduction, carbon assimilation, alkane removal, and net DIC production) for the specific alkane‐degrading groups (SCA1, SCA2 and LCA2) in marine seeps in comparison to ex‐situ measured bulk sulphate reduction (SR) and anaerobic oxidation of methane (AOM) rates.

Habitat	Station	Depth (cm)	(nmol cm^−3^ d^−1^)		SCA1‐specific rates^b^				SCA2‐specific rates^b^				LCA2‐specific rates^b^				Sum of SR rates of SCA1, SCA2, LCA2	
					(nmol cm^−3^ d^−1^)				(nmol cm^−3^ d^−1^)				(nmol cm^−3^ d^−1^)					
			Bulk SR rate^a^	Bulk AOM rate^a^	Carbon assimil.	Alkane removal	Net DIC prod.	SR	Carbon assimil.	Alkane removal	Net DIC prod.	SR	Carbon assimil.	Alkane removal	Net DIC prod.	SR	(nmol cm^−3^ d^−1^)	(% of non‐methane dependent bulk SR rates)
Northern Gulf of Mexico	GoM156	3	95	38	0.6	1.1	3.6	3.1	0.5	0.9	3.2	2.7	NA	NA	NA	0.7	6.5	11
	GoM161	5	NA	NA	0.5	0.9	3.1	2.6	0.6	1	3.4	2.9	0.3	0.2	1.9	0.7	6.2	NA
Southern GoM Chapopote Asphalt Volcano	GoM140	1	1	1	0.4	0.7	2.5	2.1	NA	NA	NA	NA					2.1	NA
	GeoB10619‐13	0	NA	NA	7.5	7.5	44.9	38.3	5.4	9.5	32.6	27.9	1.6	0.9	9.4	7.6	73.8	NA
	GeoB10619‐6	1.25	38	3	31.9	55.7	191.1	163.0	1.4	2.4	8.2	7	0.5	0.3	2.8	2.3	172.3	489
	GeoB10625‐16	0	NA	NA	0.4	0.7	2.3	2									2.0	NA
	GeoB10625‐9	3.75	404	NA	0.8	1.4	4.8	4.1	NA	NA	NA	4.7	NA	NA	NA	1.4	10.2	NA
		13.75	7	NA	1.5	2.6	9	7.7	NA	NA	NA	8.9	NA	NA	NA	0.9	17.5	NA
Guaymas Basin	GB4489‐1	0.5	NA	NA					5.1	8.9	30.4	25.9	0.5	0.3	2.9	2.4	28.3	NA
		2.5	NA	NA	1.1	2	6.9	5.9	0.6	1	3.5	3					8.9	NA
Haakon Mosby Mud Volcano	ATL19	1.5	275	234									NA	NA	NA	1.7	1.7	4
		8.5	46	38													NA	NA
	ATL22	3.5	0	3													NA	NA
Amon Mud Volcano	Amon MV760	2.5	37	4	0.7	1.3	4.4	3.8	0.2	0.3	1	0.9	0.1	0.1	0.7	0.5	5.2	16
	Amon MV825	0.5	519	61	1.1	1.9	6.6	5.6	0.2	0.3	1.2	1					6.6	1
		4.5	1321	205	4.4	7.7	26.4	22.5					0.03	0.02	0.2	0.2	22.7	2
Hydrate Ridge	HR19	4.5	757	107	2.6	4.6	15.7	13.4									13.4	2
	HR38	12.5	153	389^c^	0.1	0.2	0.8	0.7	0.3	0.5	1.7	1.5					2.2	NA
Tommeliten	1274‐K1	1.5	NA	NA													NA	NA
	1274‐K2	1.5	NA	NA	5.7	10	34.3	29.3	NA	NA	NA	0	0.4	0.2	2.1	1.7	31.0	NA
	1274‐K3	1.5	NA	NA	17.1	30	102.7	87.6	7.7	13.6	46.5	39.7					127.3	NA
		5.5	NA	NA	3.2	5.5	18.9	16.1	3.4	6	20.6	17.6					33.7	NA
		8	3^c^	1^c^					0.8	1.4	4.7	4					4.0	202

Abbreviation: NA, sample not analysed.

^a^ Ex situ bulk SR and AOM rates taken from: Orcutt et al. (2010), Wegener pers. communication, Niemann et al. (2005), Felden et al. (2013), Treude et al. (2003).

^b^ No values are given if a sample was analysed but no target cells were detected.

^c^ Rates obtained from sites close by.

Summing up the extrapolated sulphate‐reduction rates for SCA1, SCA2, and LCA2 at the investigated seep sites and comparing these values with sulphate reduction rates determined ex situ (corrected by excluding AOM‐driven sulphate reduction rates), the contribution of these groups was in the range of 1% up to 16% (Table 3). Exceptions were two sites at Chapopote Asphalt Volcano and one site at Tommeliten, where the estimated contribution exceeded 100% (Table 3). For Guaymas Basin, the contribution could not be determined since ex situ sulphate reduction rates are not available.

The rate constants constrained by the model (Table 1) were further used to estimate the contribution of SCA1, SCA2, and LCA2 to carbon assimilation, alkane removal, and DIC production in the different seep sediments. Estimated seep‐specific carbon assimilation rates were as high as 31.9 nmol cm^−3^ d^−1^ by SCA1 at the Asphalt Volcano (site GeoB10619‐6), 7.7 nmol cm^−3^ d^−1^ by SCA2 at Tommeliten (site 1274‐K3), and 1.6 nmol cm^−3^ d^−1^ by LCA2 at the Asphalt Volcano (site GeoB10619‐13) (Table 3). The extrapolated rates of alkane removal and net DIC production had their maxima at the same sites since their values are directly related to the alkane assimilation rates, with up to 55.7 nmol alkane removed and 191.1 nmol DIC produced per cm^3^ of sediment per day by SCA1 at the Asphalt Volcano (site GeoB10619‐6) (Table 3).

## 4 Discussion

### 4.1 Alkane Turnover Rates by Sulfate Reducers

Stable‐isotope probing with ^13^C‐labelled butane and dodecane revealed four major clades of sulphate‐reducing bacteria responsible for alkane degradation in Amon MV and Guaymas Basin seep sediments (Kleindienst et al. 2014). Here we show on a single‐cell level that members of these clades actively utilise butane or dodecane in these sediments. Clade SCA1 comprises organisms from different seep environments, including *Desulfosarcina aeriophaga* BuS5, the first described butane degrader isolated from Guaymas Basin sediments (Kniemeyer et al. 2007), as well as members of clade But12‐GMe, closely related organisms enriched from Gulf of Mexico seep sediments (Jaekel et al. 2013).

NanoSIMS‐based carbon assimilation rates calculated from the ^13^C enrichment in individual SCA1 cells (0.42 fmol C cell^−1^ d^−1^) were two orders of magnitude higher than the rate formerly reported for a single SCA1 member, the phylotype But12‐GMe (Jaekel et al. 2013). This was likely due to the conditions employed here, which aimed to emulate in situ settings (i.e., sediment slurry incubations in our study vs. sediment‐free enrichment cultures in the former study by Jaekel et al. 2013). Besides the presence of growth factors (e.g., vitamins and trace elements) in the source sediment, the presence of sediment particles likely played a major role. They act as a substratum for cell attachment. It has been documented that benthic bacteria prefer a surface‐attached lifestyle with > 90% of the cells living loosely or firmly attached to grain surfaces (Moncada et al. 2024). This is even more prominent for sulphate reducers, which were preferentially found as firmly attached cell fractions (Moncada et al. 2024), a feature inherently absent in sediment‐free enrichment cultures (Jaekel et al. 2013).

Cellular rates of butane consumption by SCA1 (0.73 fmol cell^−1^ d^−1^) and SCA2 (2.11 fmol cell^−1^ d^−1^) were substantially higher than those for dodecane consumption by LCA2 (0.023 fmol cell^−1^ d^−1^) (Table 1). One factor responsible for these differences is probably the lower solubility of dodecane in seawater (ca. 20 nM) compared to butane (ca. 1 mM) and therefore a lower bioavailability. Generally, the solubility of hydrocarbons in water, which is decreasing with increasing chain lengths, influences their degradation rate in marine contaminated sediments (Grossi et al. 2002).

### 4.2 SCA1 and SCA2 Contribute Substantially to Alkane Degradation

NanoSIMS analysis corroborated previous SIP results (Kleindienst et al. 2014) that showed SCA1 and SCA2 as important and active alkane degraders in the investigated sediments. Based on the applied model, SCA1 and SCA2 were responsible for about 30% and 36%, respectively, of alkane removal in our incubations (Figure 1A). They likely comprise organisms from different genera (95%–100% intragroup 16S rRNA gene sequence similarity in SCA1) or species (> 98.7% intragroup similarity in SCA2), respectively (Yarza et al. 2014). It is noteworthy that members of a single genus, namely SCA2, accounted for a substantial portion of alkane turnover in these seep sediments harbouring a highly diverse microbial community. In contrast to the highly active SCA1 and SCA2 groups, LCA2 was less active but was still responsible for about 7% of dodecane removal in Guaymas Basin sediments based on our present data (Figure 1A).

### 4.3 Additional Potential Key Players for Alkane Degradation in the Incubations

Our modelling analysis of the experimental data suggests that, in addition to our target alkane‐degrading groups, other microbes were involved in the degradation and assimilation of alkanes during the incubation experiments. Potential candidates are other SRB of the re‐classified class *Deltaproteobacteria* or archaea. Indeed, cell abundances detected by the probe set Delta495a‐c in the Guaymas Basin incubation increased approximately 6‐fold more than those of SCA2 (Table S2). The probe set Delta495a‐c targets 87% of all sequences within the *Desulfobacterota* clade (based on a probe match against the SILVA database 138.1_SSURef_NR99); therefore, encompassing the majority of the huge diversity of alkane‐degrading, sulphate‐reducing bacteria commonly found at hydrocarbon seep sites (Dhillon et al. 2003; Orcutt et al. 2010; Kleindienst et al. 2012). Furthermore, different clades of anaerobic alkane‐degrading archaea have been recently retrieved from the Guaymas Basin (for review, see Musat et al. 2024). We here detected the thermophilic, butane‐oxidising *Ca*. Syntrophoarchaeum (Laso‐Pérez et al. 2016) as well as the ethane‐oxidising *Ca*. Argoarchaeum and *Ca*. Ethanoperedens (Chen et al. 2019; Hahn et al. 2020) that were described to live in syntrophy with sulphate‐reducing partner bacteria of the *Desulfofervidus*/HotSeep 1 and *Desulfosarcina/Desulfococcus* clades. Although quantification of these groups was not possible due to the presence of only few but large cell aggregates, their detection in this study suggests a potential contribution to alkane consumption in our incubations. Possible archaeal candidates for long‐chain alkane degradation could be *Ca*. Alkanophaga, a thermophilic C5 to C14 alkane degrader that lives in syntrophy with sulphate‐reducing *Ca*. Thermodesulfobacterium syntrophicum (Zehnle et al. 2023), or *Ca*. Methanoliparia, a non‐syntrophic methanogenic C13‐C39 alkane degrader (Zhou et al. 2022). Nevertheless, our incubation temperature conditions of 28°C for Guaymas Basin sediments were not optimal for all the archaeal clades mentioned above, which were described either as thermophiles or as psychrophiles. If these abundant clades consumed substantial parts of the butane or dodecane in our incubations, their identification via stable‐isotope probing would have been challenging because of their slow growth (doubling times of weeks to months) and expected minimal assimilation of ^13^C into their biomass. Indeed, in our previous study, we did not find any indications for label incorporation by archaea (Kleindienst et al. 2014).

The broader phylogenetic diversity of bacteria and archaea involved in anaerobic degradation of alkanes other than methane contrasts with the relatively conserved ANME lineages involved in AOM (Wegener et al. 2022) highlighting a substantial potential of a diverse microbial community for higher alkane degradation in seep sediments. The yet undetected alkane degraders of the re‐classified class *Deltaproteobacteria* and/or archaea and/or other bacterial clades in the incubations remain to be addressed in future studies, for example by cultivation strategies or stable‐isotope probing combined with metagenomics (Chen and Murrell 2010).

### 4.4 Model Predictions and Their Uncertainty

We used a mass‐balance model to quantitatively interpret the diverse data collected in the present study. This approach provided several valuable insights, as discussed in the following. First, the model enabled us to constrain the rates of cellular activity of the target groups (Table 1) and provided clarity regarding their interpretation. More specifically, the rates derived from the present data represent maximal estimates of cellular activity associated with alkane degradation (i.e., alkane uptake, SR, and DIC production). These estimates correspond to the rates that would occur if the cells experienced no substrate limitation and grew exponentially under the conditions present in the sediment slurry, including the alkane added in excess. Such rates occurred at the beginning of the incubations but gradually declined over time. This decline was unlikely due to substrate (alkane) limitation. Instead, it was caused by another, non‐specific limiting factor, as supported by the cell counts data being adequately described by a logistic growth model alone (i.e., without a substrate limitation term).

Another valuable insight gained through the modelling analysis is that additional microbial groups, different from those targeted in the present study, may significantly contribute to alkane degradation in the studied sediments. This suggestion arose from a mismatch in carbon balances identified when comparing carbon concentrations (total C and ^13^C) obtained from bulk measurements with those predicted by the model through upscaling of the single‐cell data.

We emphasise that the confidence in our final rate estimates is limited by several factors, the most important of which are discussed in the following. First, we analysed about 10 to 60 cells per time point, which is reasonable given the challenges associated with the identification and NanoSIMS analysis of cells isolated from sediment slurries. However, some target cells formed aggregates where the variation in activity among cells was substantially lower than that observed among aggregates (Figure 3). Consequently, our rate estimates are likely influenced by undersampling.

Second, cell abundances and biovolumes were determined based on CARD‐FISH, which is a robust method for in situ enumeration of microorganisms (Amann and Fuchs 2008). However, biovolumes derived based on the measured cell sizes after CARD‐FISH result in an overestimation, which can be expected to be approximately 1.4‐fold of the actual cell volume (A. Ellrott, MPI Bremen, personal communication; preliminary data). Additionally, the number of cells measured may not be sufficiently high for robust statistics and to overcome the high variability in cell sizes observed. Furthermore, some cells from the target groups might have been undetected due to insufficient probe coverage.

Third, we used the carbon density of *Desulfosarcina aeriophaga* BuS5, a member of clade SCA1, as an estimate for the carbon density of all target groups, that is, SCA1, SCA2, and LCA2. It is likely that the carbon densities of the individual groups are significantly different from each other.

Finally, for the GB‐dodecane incubation, the activity of secondary metabolite consumers likely contributed to the discrepancy between model predictions and experimental data. Our previous stable‐isotope probing study (Kleindienst et al. 2014) identified distinct bacterial groups as potential secondary consumers, that is, microbes that used labelled byproducts or dead biomass from the primary consumers as a substrate. These secondary consumers were identified as uncultured *Bacteroidota*, uncultured *Desulfobacteraceae*, as well as *Ca*. Omnitrophica (OP3) (Kleindienst et al. 2014). It is likely that these microbes contributed to the production of ^13^C‐TOC at later time points, which is not captured by the present model, in particular for the incubation with labelled dodecane (Figure 3C). Nevertheless, excluding secondary consumers from the model did not influence our rate estimates for the target cells.

### 4.5 Major Impact of SCA1, SCA2, and LCA2 on Sulfate Reduction in Hydrocarbon Seep Sediments

The extrapolation of sulphate reduction rates for SCA1, SCA2, and LCA2 indicated that these specific clades have the potential to substantially contribute to hydrocarbon degradation processes at marine hydrocarbon seeps. A comparison of the extrapolated values with ex situ rate measurements from Chapopote asphalt volcano at Gulf of Mexico suggested that all of the sulphate reduction not driven by AOM may be mediated by SCA1, SCA2, and LCA2 in certain sediment depth horizons (Table 3). Additionally, previous studies from similar seep sites in the Gulf of Mexico suggested that complex hydrocarbon mixtures fuel a diverse SRB community, leading to high sulphate reduction rates that are coupled to hydrocarbon turnover (Orcutt et al. 2010; Bowles et al. 2011). As expected, at Amon MV, which is the source of some of our sediment samples, a substantial portion of in situ bulk sulphate reduction (up to 16%) could be attributed to SCA1, SCA2, and LCA2. This finding further supports a biogeochemical relevance of these clades, complementing previous studies that showed microbial degradation of gaseous alkanes at this site (Felden et al. 2013). In contrast, the potential of SCA1, SCA2, and LCA2 for non‐AOM driven sulphate reduction was estimated to be rather low (up to 4%) in cold sediments that are mainly influenced by methane seepage, for example, at the Haakon Mosby MV or Hydrate Ridge (Table 3). Here, other clades of butane or dodecane degraders could be responsible for a large part of AOM‐independent SR that cannot be explained by SCA1, SCA2, or LCA2. Valentine et al. (2005) proposed a dissolved organic carbon‐based heterotrophy in methane seep environments, particularly at high‐flux seeps covered by microbial mats of chemolithoautotrophic sulphide‐oxidising bacteria. The coupling of SR to the oxidation of dissolved organic matter may account for much of the unexplained SR observed in such environments.

It is also important to note that the sediments used for ex situ rate measurements and for CARD‐FISH did not derive from exactly the same spot and that some of the sediments used for cell counting were covered by a microbial mat, resulting in higher cell numbers. Therefore, any comparison between the ex situ rates and the extrapolated rates should be interpreted with caution. As mentioned above, the rates presented in this study, including cell‐specific and bulk sediment‐specific rates of alkane removal and sulphate reduction, can be considered near‐maximal estimates. The alkane degraders in our incubations were not exposed to substrate or electron acceptor limitation, as often encountered in the environment. The initial alkane concentrations were 100 to 1000 times higher than those typically found in marine sediments (e.g., Kleindienst et al. 2014; M. Kellermann, University of Oldenburg, pers. communication). In addition, sulphate was homogeneously mixed throughout the slurries, eliminating diffusion‐based limitations. This contrasts with subseafloor environments like the Gulf of Mexico, where sulphate diffusion alone is considered insufficient to sustain high SR rates (Bowles et al. 2011).

## 5 Conclusion

Clades SCA1, SCA2, and LCA2 are important key players for sulphur and carbon cycling at hydrocarbon seeps. Our extrapolated SR rates indicate that these specific clades have the potential to substantially contribute to SR at seep sites dominated by non‐methane hydrocarbons. We have demonstrated that our methodological approach, which combines stable isotopes labelling experiments with single‐cell analyses by Nano‐SIMS and FISH, can be successfully applied to challenging samples from complex habitats such as marine sediments. Using a similar approach, future research could investigate, for instance, the contribution of recently identified syntrophic and free‐living archaeal hydrocarbon degraders under high temperature conditions or the competition between syntrophic hydrocarbon‐degrading archaea and free‐living hydrocarbon‐degrading bacteria.

## Author Contributions

**Sara Kleindienst:** investigation (incubations, NanoSIMS, cell counts), data analysis, data interpretation, visualization, writing – original draft, writing – review and editing. **Lubos Polerecky:** methodology, investigation (modeling), data analysis, data interpretation, visualization, writing – original draft, writing – review and editing, validation. **Rudolf Amann:** conceptualization, writing – review and editing, funding acquisition, resources. **Florin Musat:** conceptualization, supervision, investigation (modeling), data analysis, data interpretation, data curation, writing – original draft, writing – review and editing, validation. **Katrin Knittel:** conceptualization, supervision, data interpretation, project administration, writing – original draft, writing – review and editing, validation, funding acquisition.

## Conflicts of Interest

The authors declare no conflicts of interest.

## Supporting information


**DATA S1.** Supporting Information.

## Data Availability

The data that supports the findings of this study are available in the Supporting Information of this article.
