# Supplementary material for: In Situ Metabolic Rates of Alkane‐Degrading Sulphate‐Reducing Bacteria in Hydrocarbon Seep Sediments Revealed by Combining CARD‐FISH, NanoSIMS, and Mathematical Modelling
Source: Environ Microbiol. 2025 Jul 29;27(8):e70151. doi: 10.1111/1462-2920.70151 (PMC12306147; doi:10.1111/1462-2920.70151)
Supplement: Supplementary file 1 — DATA S1. Supporting Information. [file EMI-27-e70151-s001.pdf]

Supplementary Material

In situ metabolic rates of alkane-degrading sulfate-reducing bacteria in hydrocarbon seep sediments revealed by combining CARD-FISH, NanoSIMS, and mathematical modeling

Sara Kleindienst[1,2,*], Lubos Polerecky[1,3], Rudolf Amann[1], Florin Musat[1,4], Katrin Knittel[1,*]

[1]Max Planck Institute for Marine Microbiology, Bremen, Germany

[2]Department of Environmental Microbiology, Institute for Sanitary Engineering, Water Quality and Solid Waste Management (ISWA), University of Stuttgart, Stuttgart, Germany

[3]Department of Earth Sciences, Faculty of Geosciences, Utrecht University, Utrecht, The Netherlands

[4]Department of Biology, Section for Microbiology, Aarhus University, Aarhus, Denmark

Supplementary Methods

Model of alkane assimilation and dissimilation by the target cells

Here we formulate mathematical equations for tracking the transformations of carbon in a growing population of alkane degrading cells. Of specific interest is the flow of the ^{13}C and ^{12}C isotopes between the carbon source (dissolved alkane and inorganic carbon) and the target cell biomass during our incubations with ^{13}C-labeled alkane.

Growth of the target cell population

Experimental data showed that the cell counts of the target cells, N, increased during the incubation. To model the growth of the target cells, we assumed that the increase in the number of cells was described by the differential equation

$$\frac{dN}{dt} = k \cdot N \cdot f_{\mathrm{lim}} \qquad \text{(Eq. S1)}$$

where f_{lim} is a factor describing growth limitation ($f_{\mathrm{lim}} \leq 1$), and k is the rate constant (in d^{-1}). The rate constant describes the situation when the cells grow *exponentially* ($N = N_{\mathrm{ini}} \cdot \exp(k \cdot t)$), which occurs in the absence of limitation (i.e., when $f_{lim} = 1$). In our model we assume that the growth limitation factor is a product of three contributions, $f_{\mathrm{lim}} = f_{\mathrm{sub}} \cdot f_{\mathrm{lag}} \cdot f_{\mathrm{stat}}$. The first contribution, $f_{\mathrm{sub}} = A/(A+K_A)$, describes the growth limitation by substrate (here the added alkane) according to the Michaelis-Menten kinetics, where A is the alkane concentration and K_A is the substrate affinity constant. The other two contributions, $f_{\mathrm{lag}} = q/[q+\exp(-k \cdot t)]$ and $f_{\mathrm{stat}} = (1-N/N_{\mathrm{max}})$, account for substrate non-specific limitations involved during the lag and stationary phases in the growth of bacterial populations, respectively, as proposed by Baranyi et al., (1993; 1994). In these expressions, the parameters q and N_{max} are related, respectively, to the duration of the lag phase and the onset of the stationary phase (note: in models of population dynamics N_{max} is referred to as the carrying capacity of the system, i.e., the maximum size of the population the system can support).

From cell counts to carbon content

To convert the cell counts into units of carbon, we assumed that the carbon content of an average individual target cell is C_{cell}. Thus, the carbon content of a population of N of such cells is $C = N \cdot C_{\mathrm{cell}}$. Assuming that the carbon content of an average target cell during the incubation was constant, equation S1 implies the following differential equation for the carbon content of the target population:

$$\frac{dC}{dt} = k \cdot C \cdot f_{\mathrm{lim}}. \qquad \text{(Eq. S2)}$$

Carbon mass balance

In our incubations, the growth of the target cells was driven by the ^{13}C-labeled alkane, which served both as the carbon source and energy source. We assumed that part α of the alkane pool was assimilated, leading to the increase in the target cells biomass, while the remaining part 1−α was used as an energy source, leading to the production of dissolved inorganic carbon (DIC). Thus, for an infinitesimal amount of alkane removed from the alkane pool, d*A* (d*A*<0), the amount of carbon assimilated into the target cells biomass is

$$dC_{\text{assim\_from\_alkane}} = -\alpha \cdot \nu_{\text{alkane}} \cdot dA, \qquad \text{(Eq. S3)}$$

and the amount of DIC produced is

$$dC_{\text{DIC\_from\_alkane}} = -(1-\alpha) \cdot \nu_{\text{alkane}} \cdot dA, \qquad \text{(Eq. S4)}$$

where the stoichiometric coefficient ν_{alkane} is 4 for butane (C_4H_{10}) and 12 for dodecane ($C_{12}H_{26}$).

In addition to carbon originating from alkane, the target cells also assimilate inorganic carbon (i.e., via carboxylation reactions). We assumed that during the incubation the alkane and DIC pools were the only carbon sources utilized by the target cells for growth. Thus, we denoted the parts of the assimilated carbon originating from the alkane and DIC pool by β and 1−β, respectively. This implies that, for an infinitesimal amount of *total* assimilated carbon, d*C*, the corresponding infinitesimal amounts of carbon assimilated from the alkane and DIC pools are

$$dC_{\text{assim\_from\_alkane}} = \beta \cdot dC, \qquad \text{(Eq. S5)}$$

$$dC_{\text{assim\_from\_DIC}} = (1-\beta) \cdot dC. \qquad \text{(Eq. S6)}$$

During the incubation, the inorganic carbon was both produced (through alkane dissimilation) and consumed (due to assimilation into the target cells biomass). We assumed that alkane dissimilation and DIC assimilation were the only source and sink of DIC during the incubation, respectively. This implies that the *net* infinitesimal amount of carbon added to the DIC pool by these two processes is

$$dC_{\text{DIC}} = dC_{\text{DIC\_from\_alkane}} - dC_{\text{assim\_from\_DIC}}. \qquad \text{(Eq. S7)}$$

Using above equations, we now derive explicit relationships between the changes in the carbon contents of the carbon pools involved. First, by combining equations S3−S6 with equation S7, we obtain the following relationship between the total carbon assimilated into the target cells biomass and the net-produced DIC:

$$dC_{DIC} = \left[\frac{\beta}{\alpha} - 1\right] \cdot dC. \qquad \text{(Eq. S8)}$$

Second, by combining equations S3 and S5, we obtain the following relationship between the total carbon assimilated into the target cells biomass and the amount of removed alkane:

$$dA = -\frac{\beta}{\alpha \nu_{\text{alkane}}} \cdot dC. \qquad \text{(Eq. S9)}$$

Finally, by dividing equations S8−S9 by an infinitesimal time step dt, we obtain the following relationships between the rates of alkane removal (dA/dt), DIC production (dC_{DIC}/dt), and biomass production of the target cells (dC/dt):

$$\frac{dA}{dt} = -\frac{\beta}{\alpha \nu_{\text{alkane}}} \cdot \frac{dC}{dt}, \qquad \text{(Eq. S10)}$$

$$\frac{dC_{\text{DIC}}}{dt} = \left[\frac{\beta}{\alpha} - 1\right] \cdot \frac{dC}{dt}. \qquad \text{(Eq. S11)}$$

Cellular rates

The term dC/dt in the above equations represents the rate of change in the *total* carbon content of the target population (in units of mol C d^{-1}). By dividing this term with the number of target cells, N, we obtain the rate of change in the carbon content of an *average* target cell, i.e., the *cellular carbon uptake rate* by an average target cell. We denote this parameter as r_C (in units of mol C d^{-1} $cell^{-1}$). Equation S2 implies that the cellular carbon uptake rate and the rate constant k are related according to

$$r_C = k \cdot C_{\text{cell}} \cdot f_{\text{lim}}. \qquad \text{(Eq. S12)}$$

If the growth of the target cells is *not* limited, which occurs when the rate limitation factor f_{lim} = 1, the cellular carbon uptake rate reaches a maximum value of

$$r_C^{\text{max}} = k \cdot C_{\text{cell}}. \qquad \text{(Eq. S13)}$$

Thus, for a given rate constant k and cellular carbon content C_{cell}, the product $k \cdot C_{\text{cell}}$ represents the *maximal cellular carbon uptake rate*, i.e., the rate at which an average cell from an exponentially growing cell population would assimilate carbon.

Similar parameters can be identified with respect to the consumption of alkane and the net production of DIC. Specifically, by dividing equations S10 and S11 with N, we obtain the following expressions for the maximal cellular rate of alkane consumption (in mol alkane d^{-1} $cell^{-1}$) and the maximal cellular rate of net DIC production (in mol C d^{-1} $cell^{-1}$):

$$r_{\text{alkane}}^{\text{max}} \equiv -\frac{1}{N} \cdot \frac{dA}{dt} = \frac{\beta}{\alpha \cdot \nu_{\text{alkane}}} \cdot k \cdot C_{\text{cell}}, \qquad \text{(Eq. S14)}$$

$$r_{\text{DIC}}^{\text{max}} \equiv \frac{1}{N} \cdot \frac{dC_{\text{DIC}}}{dt} = \left[\frac{\beta}{\alpha} - 1\right] \cdot k \cdot C_{\text{cell}}. \qquad \text{(Eq. S15)}$$

In addition to the production of DIC, sulfate-reducing bacteria (e.g., the target cells) couple the oxidation of alkanes to the reduction of sulfate leading to the production of hydrogen sulfide (H_2S). The ratio between the stoichiometric coefficients of the produced H_2S and DIC is equal to ν_{H2S}/ν_{alkane}, where $\nu_{H2S} = (3\nu_{alkane} + 1)/4$ (see Methods). Therefore, the maximal cellular rate of H_2S production, which is the same as the maximal cellular rate of sulfate reduction (in mol S d^{-1} $cell^{-1}$), is

$$r_{\mathrm{SR}}^{\mathrm{max}} = \frac{1-\alpha}{\alpha} \cdot \beta \cdot \frac{\nu_{H_2S}}{\nu_{\mathrm{alkane}}} \cdot k \cdot C_{\mathrm{cell}}. \qquad \text{(Eq. S16)}$$

Differential equations for the ^{12}C and ^{13}C isotopes of the target cells, DIC and alkane pools

Based on the differential equations S2, S10 and S11 for the total carbon contents of the target population (C), alkane (A) and DIC (C_{DIC}), we now formulate differential equations for the corresponding ^{12}C and ^{13}C isotopes. Because the carbon assimilated by the target cells originates from the alkane and DIC pools, the increase in the ^{13}C isotope of the target cells has two contributions. We assume that kinetic isotope effects are negligible; thus, we assume that each contribution is proportional to the ^{13}C atom fraction of the respective carbon source. We denote the ^{13}C atom fractions (i.e., the molar ratio $^{13}C/(^{12}C+^{13}C)$) of the alkane and DIC pools as $X(^{13}C)_{alkane}$ and $X(^{13}C)_{DIC}$, respectively. Thus, taking into account equations S5 and S6, the rate of increase in the ^{13}C isotope of the target cells biomass is

$$\frac{d\,^{13}C}{dt} = \left[\beta \cdot X\left(^{13}C\right)_{\mathrm{alkane}} + (1-\beta) \cdot X\left(^{13}C\right)_{\mathrm{DIC}}\right] \cdot k \cdot C \cdot f_{\mathrm{lim}}. \qquad \text{(Eq. S17)}$$

Similarly, based on equation S7, the rate of increase in the ^{13}C isotope of the DIC pool is

$$\frac{d\,^{13}C_{\mathrm{DIC}}}{dt} = \left[\frac{1-\alpha}{\alpha} \cdot \beta \cdot X\left(^{13}C\right)_{\mathrm{alkane}} - (1-\beta) \cdot X\left(^{13}C\right)_{\mathrm{DIC}}\right] \cdot k \cdot C \cdot f_{\mathrm{lim}}. \qquad \text{(Eq. S18)}$$

The same argument applies to the ^{12}C isotope, except the contributions are proportional to the ^{12}C atom fractions (i.e., the molar ratio $^{12}C/(^{12}C+^{13}C)$). Thus, the rates of increase in the ^{12}C isotope of the target cells biomass and of the DIC pool are, respectively,

$$\frac{d\,^{12}C}{dt} = \left[\beta \cdot X\left(^{12}C\right)_{\mathrm{alkane}} + (1-\beta) \cdot X\left(^{12}C\right)_{\mathrm{DIC}}\right] \cdot k \cdot C \cdot f_{\mathrm{lim}} \qquad \text{(Eq. S19)}$$

$$\frac{d\,^{12}C_{\mathrm{DIC}}}{dt} = \left[\frac{1-\alpha}{\alpha} \cdot \beta \cdot X\left(^{12}C\right)_{\mathrm{alkane}} - (1-\beta) \cdot X\left(^{12}C\right)_{\mathrm{DIC}}\right] \cdot k \cdot C \cdot f_{\mathrm{lim}} \qquad \text{(Eq. S20)}$$

Note that the ^{12}C and ^{13}C atom fractions are related according to $X(^{12}C) = 1-X(^{13}C)$.

We assumed that alkane was only *consumed* during the incubation. Therefore, the differential equations for the removal of the ^{13}C and ^{12}C isotopes from the alkane pool are (see Eq. S10)

$$\frac{d^{13}A}{dt} = -\frac{\beta}{\alpha \cdot \nu_{\text{alkane}}} \cdot X\left(^{13}C\right)_{\text{alkane}} \cdot k \cdot C \cdot f_{\text{lim}}, \quad \text{(Eq. S21)}$$

$$\frac{d^{12}A}{dt} = -\frac{\beta}{\alpha \cdot \nu_{\text{alkane}}} \cdot X\left(^{12}C\right)_{\text{alkane}} \cdot k \cdot C \cdot f_{\text{lim}}. \quad \text{(Eq. S22)}$$

Solution of the differential equations

Differential equations S2 and S17−S22 form the mathematical basis for the quantification of the carbon flow between the different carbon pools during the incubation. In general, they are non-linear because the rate limitation factors f_{stat} and f_{sub} depend on the carbon contents of the target cells (C) and alkane (A), respectively, and time-dependent because the factor f_{lag} depends on time. Therefore, we solved them numerically, using R (R Core Team 2024) and the R-package deSolve (Soetaert et al., 2010).

For our incubation experiment we assumed the following initial conditions: (i) initial carbon content of the target cells biomass, C_{ini}, calculated as $C_{\text{ini}} = N_{\text{ini}} \cdot C_{\text{cell}}$, where N_{ini} is the initial target cell count (estimated from fitting the measured cell counts) and C_{cell} is the average carbon content of an individual target cell (estimated from the measured biovolume); (ii) initial ^{13}C atom fraction of the target cells, $X(^{13}\text{C})_{\text{ini}}$ (measured); (iii) initial carbon content of the DIC pool, $C_{\text{DIC,ini}}$ (estimated from the measured ^{13}C-DIC data); (iv) initial ^{13}C atom fraction of the DIC pool, $X(^{13}\text{C})_{\text{DIC,ini}}$ (measured); (v) initial amount of the added alkane, A_{ini} (measured); (vi) initial ^{13}C atom fraction of the added alkane, $X(^{13}\text{C})_{\text{alkane,ini}} = 0.99$ (nominal value given by the manufacturer).

^{12}C and ^{13}C isotopes in the total organic carbon pool

In addition to the ^{12}C and ^{13}C isotopes for the carbon pools of the target population, DIC and alkane, we also determined the isotopes for the total organic carbon (TOC) pool in the sediment slurries. We assumed that the increase in ^{13}C-TOC was only due to the assimilation of the ^{13}C labeled alkane and DIC by the target cells, while other possible processes, e.g., uptake of labeled byproducts or dead biomass by secondary consumers or autotrophic fixation of the ^{13}C-enriched DIC by other organisms, were ignored. Thus, the ^{13}C atom fraction in the TOC pool was calculated according to

$$X\left(^{13}C\right)_{\text{TOC}} = \frac{X\left(^{13}C\right)_{\text{TOC,ini}} \cdot C_{\text{TOC,ini}} + {}^{13}C}{C_{\text{TOC,ini}} + {}^{13}C + {}^{12}C}, \quad \text{(Eq. S23)}$$

where $C_{\text{TOC,ini}}$ is the initial carbon content in the TOC pool (measured), $X(^{13}\text{C})_{\text{TOC,ini}}$ is the initial ^{13}C atom fraction in the TOC pool (measured), and ^{13}C and ^{12}C are the carbon contents of the target population (calculated by solving the differential equations S2 and S17−S22; see above).

Supplementary Tables and Figures

Table S1: Oligonucleotide probes used for CARD-FISH.

Probe name	Specificity	Formamide [%]	Sequence (5' to 3')	Reference
Delta495a-c[▼$]	Most *Desulfobacterota, Bdellovibrionota, some Myxococcota* and clades NB1-j and Sva0485, outgroup hits in *Gemmatimonadota*, *Planctomycetota*, *Cyanobacteria*, *Firmicutes*[Δ]	30	AGT TAG CCG GTG CTT CCT<br>AGT TAG CCG GCG CTT CCT<br>AAT TAG CCG GTG CTT CCT	(Loy et al., 2002)
cDelta495a-c	Competitors		AGT TAG CCG GTG CTT CTT<br>AGT TAG CCG GCG CTT CKT<br>AAT TAG CCG GTG CTT CTT	(Macalady et al., 2006)<br>(Lücker et al., 2007)<br>(Lücker et al., 2007)
DSS658	*Desulfosarcina/ Desulfococcus* (DSS) branch	50	TCC ACT TCC CTC TCC CAT	(Manz et al., 1998)
cDSS658	competitor		TCC ACT TCC CTC TCC GGT	(Kleindienst et al., 2012)
SCA1-212a/b	SCA1 clade	20	CAT CCC MAA ACA GTA GCT	(Kleindienst et al., 2014)
SCA2-138	SCA2 clade	25	CGA GTT ATC CCC GAT TCG	(Kleindienst et al., 2014)
LCA2-63	LCA2 clade	10	GCU AAA GCU UUC UCG UUC	(Kleindienst et al., 2014)
Arch915	Most *Archaea*	35	GTG CTC CCC CGC CAA TTC CT	(Alm et al., 1996)

▼ probes used in a mix at equimolar concentrations.

$ probe Delta495a-c was originally designed to target the re-classified class *Deltaproteobacteria*

Δ Hits in non-target groups are reported only for taxa contributing more than 180 hits in the SILVA database 138.1_SSURef_NR99 (510,495 sequences total).

Table S2: Absolute cell abundance per mL of sediment slurry (expressed as 10^7 cells cm^{-3} slurry), for total cells, *Deltaproteobacteria, Desulfosarcina/Desulfococcus* (DSS), specific alkane-degrading clades (SCA1, SCA2, or LCA2), and archaea in NanoSIMS incubations, as determined by AODC and CARD-FISH. Each time point represents a different incubation vial. A grey background indicates vials selected for NanoSIMS analysis.

**Incubation**	**Time [d]**	**Total cells** (10^7 cm^{-3})	***Deltaprot.*** (10^7 cm^{-3})	**DSS** (10^7 cm^{-3})	**SCA1** (10^7 cm^{-3})	**SCA2** (10^7 cm^{-3})	**LCA2** (10^7 cm^{-3})	***Archaea*** (10^7 cm^{-3})
AMV-Butane	0	54.6	8.9	4.9	0.9	NA	NA	NA
	9	46.1	7.9	5.3	1.8	NA	NA	NA
	15	60.1	10.2	7.7	2.9	NA	NA	NA
	29	73.8	14.4	14.7	5.3	NA	NA	NA
GB-Butane	0	17.6	0.8	0.4	NA	0.3	NA	4.7
	29	20.0	1.6	0.7	NA	0.2	NA	11.4
	57	38.5	5.0	2.4	NA	1.0	NA	10.0
	71	31.2	3.7	2.0	NA	1.2	NA	NA
	113	41.3	7.5	2.6	NA	1.8	NA	NA
GB-Dodecane	0	17.6	0.7	0.3	NA	NA	0.0	4.7
	29	50.1	7.8	3.2	NA	NA	0.8	5.3
	115	31.9	4.6	2.1	NA	NA	2.0	7.5
	183	31.4	6.9	1.9	NA	NA	2.0	NA
	232	39.6	5.8	2.5	NA	NA	2.5	NA

NA = not analyzed

Table S3: Cell dimensions, biovolume, and carbon content of SCA1, SCA2, and LCA2 in the three incubations.

Clade	Length		Diameter		Biovolume				Carbon content[1]	
	mean [µm]	SD[2]	mean [µm]	SD[2]	mean [µm^3]	SD[2]	CV[3] %	N_{cells}	mean [fmol C cell^{-1}]	Rel. SE[4] %
SCA1 in AMV-Butane	2.10	0.26	1.10	0.11	1.66	0.42	25	25	7.6	5
SCA2 in GB-Butane	2.53	0.62	1.82	0.48	5.33	3.54	66	71	24.4	8
LCA2 in GB-Dodecane	0.96	0.10	0.96	0.10	0.48	0.17	35	24	2.2	7

[1] Calculated assuming cellular carbon density of 55 fg C µm^{-3}.
[2] Standard deviation.
[3] Coefficient of variation; CV = SD/mean.
[4] Relative standard error of the mean; Rel. SE = SD/sqrt(N_{cells})/mean.

Table S4: Biovolumes of the alkane-degrading clades SCA1, SCA2 and LCA2 in various seep sediments. Biovolumes were calculated based on cell size measurements of CARD-FISH-stained cells.

Habitat	Station	Depth	SCA1 volume (µm^3)							SCA2 volume (µm^3)							LCA2 volume (µm^3)						
		[cm]	N	Av	Stdev	Min	Max	CV (%)	SE (%)	N	Av	Stdev	Min	Max	CV (%)	SE (%)	N	Av	Stdev	Min	Max	CV (%)	SE (%)
**Northern GoM**	**156**	3	11	0.46	0.40	0.05	1.32	86%	26%	6	0.45	0.60	0.10	1.65	132%	54%	NA	NA	NA	NA	NA	NA	NA
	**161**	5	10	0.32	0.14	0.08	0.50	44%	14%	9	0.40	0.27	0.12	0.99	69%	23%	5	0.26	0.07	0.15	0.32	27%	12%
**Southern GoM: Chapopote Asphalt Volcano**	**140**	1	11	0.77	0.56	0.04	2.03	74%	22%	NA	NA	NA	NA	NA	NA	NA	NA	NA	NA	NA	NA	NA	NA
	**GeoB10619-13**	0	11	0.48	0.65	0.07	1.92	136%	41%	16	0.39	0.32	0.00	1.22	83%	21%	1	0.10					
	**GeoB10619-6**	1.25	10	0.79	1.13	0.08	3.88	143%	45%	7	0.43	0.25	0.05	0.80	58%	22%	3	0.40	0.22	0.25	0.65	54%	31%
	**GeoB10625-16**	0	8	0.32	0.08	0.23	0.45	24%	9%	NA	NA	NA	NA	NA	NA	NA	NA	NA	NA	NA	NA	NA	NA
	**GeoB10625-9**	3.75	8	0.32	0.08	0.23	0.45	24%	9%	NA	NA	NA	NA	NA	NA	NA	NA	NA	NA	NA	NA	NA	NA
		13.75	8	0.32	0.08	0.23	0.45	24%	9%	NA	NA	NA	NA	NA	NA	NA	NA	NA	NA	NA	NA	NA	NA
**Guaymas Basin**	**BG4489-1**	0.5	7	0.91	0.17	0.69	1.13	19%	7%	2	0.51	0.46	0.19	0.84	90%	63%	2	0.13	0.01	0.12	0.14	10%	7%
		2.5	7	0.91	0.17	0.69	1.13	19%	7%	2	0.51	0.46	0.19	0.84	90%	63%	2	0.13	0.01	0.12	0.14	10%	7%
**Haakon Mosby MV (HMMV)**	**ATL19**	1.5	3	1.33	1.04	0.40	2.45	78%	45%	NA	NA	NA	NA	NA	NA	NA	NA	NA	NA	NA	NA	NA	NA
		8.5	3	1.33	1.04	0.40	2.45	78%	45%	NA	NA	NA	NA	NA	NA	NA	NA	NA	NA	NA	NA	NA	NA
	**ATL22**	3.5	3	1.33	1.04	0.40	2.45	78%	45%	NA	NA	NA	NA	NA	NA	NA	NA	NA	NA	NA	NA	NA	NA
**Amon MV**	**AMV760**	2.5	10	0.61	0.48	0.05	1.53	79%	25%	1	0.48						8	0.27	0.11	0.12	0.41	40%	14%
	**AMV825**	0.5	17	0.86	0.50	0.36	2.00	58%	14%	2	0.17	0.05	0.14	0.21	30%	21%	7	0.26	0.14	0.12	0.56	56%	21%
		4.5	17	0.86	0.50	0.36	2.00	58%	14%	NA	NA	NA	NA	NA	NA	NA	7	0.26	0.14	0.12	0.56	56%	21%
**Hydrate Ridge**	**HR19**	4.5	11	0.55	0.40	0.14	1.26	74%	22%	1	0.30						10	0.49	0.24	0.14	0.99	48%	15%
	**HR38**	12.5	17	0.17	0.15	0.03	0.65	88%	21%	10	0.40	0.28	0.11	0.98	71%	22%	NA	NA	NA	NA	NA	NA	NA
**Tommeliten**	**1274-K1**	1.5	NA	NA	NA	NA	NA	NA	NA	3	0.96	1.43	0.14	2.61	148%	86%	3	0.34	0.23	0.09	0.53	67%	39%
	**1274-K2**	1.5	10	0.61	0.45	0.11	1.65	74%	24%	NA	NA	NA	NA	NA	NA	NA	9	0.34	0.36	0.05	1.24	107%	36%
	**1274-K3**	1.5	2	0.48	0.12	0.39	0.57	26%	19%	4	0.40	0.26	0.11	0.66	65%	32%	4	0.51	0.31	0.17	0.83	61%	30%
		5.5	2	0.48	0.12	0.39	0.57	26%	19%	4	0.40	0.26	0.11	0.66	65%	32%	4	0.51	0.31	0.17	0.83	61%	30%
		8	2	0.48	0.12	0.39	0.57	26%	19%	4	0.40	0.26	0.11	0.66	65%	32%	4	0.51	0.31	0.17	0.83	61%	30%

NA, sample not analyzed

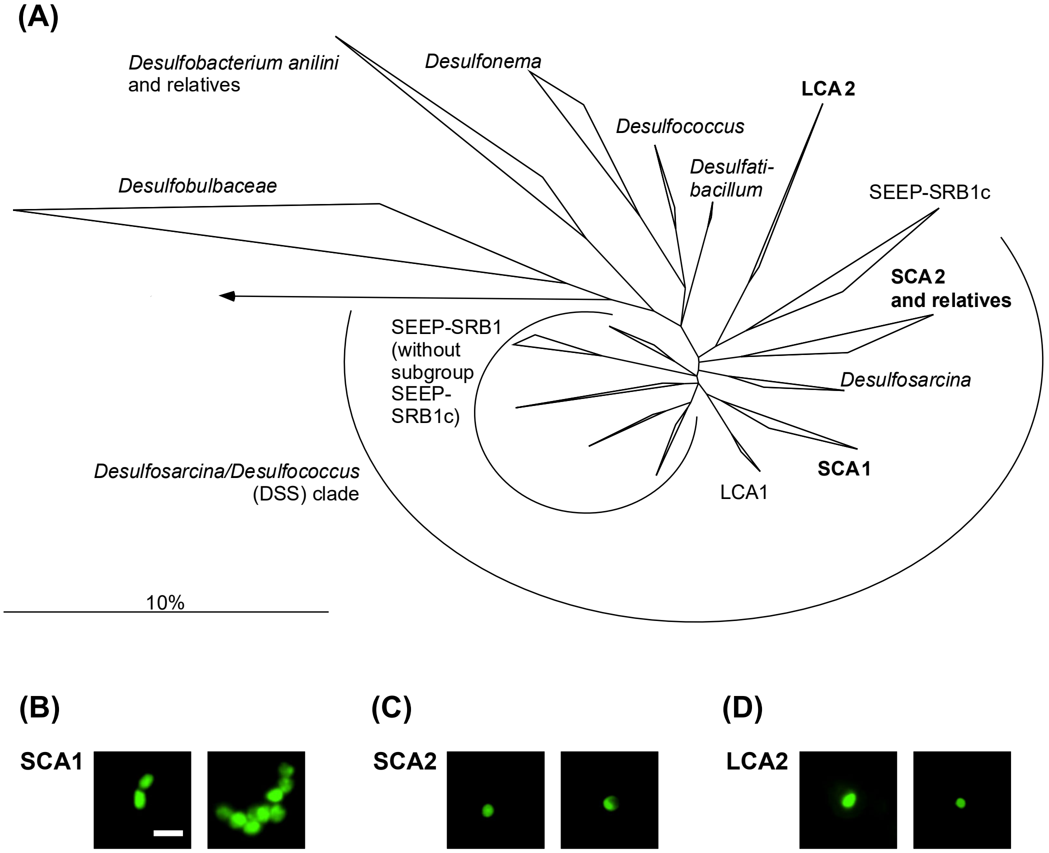

Figure S1: Phylogenetic tree based on 16S rRNA genes showing the groups SCA1 and SCA2 as well as LCA2 (bold) within *Desulfobacterota* (A). Images show SCA1 cells in sediments from the Tommeliten seeps and the Chapopote asphalt volcano (B), SCA2 cells in sediments from the Guaymas Basin and the Tommeliten seeps (C), as well as as LCA2 cells in sediments from Amon Mud Volcano and Chapopote asphalt volcano (D), visualized by CARD-FISH. Scale bar represents 2 µm for all images.

References

Alm, E.W., Oerther, D.B., Larsen, N., Stahl, D.A., and Raskin, L. (1996) The oligonucleotide probe database. *Appl Environ Microbiol* **62**: 3557-3559.

Baranyi, J., and Roberts, T.A. (1994) A dynamic approach to predicting bacterial growth in food. *Int J Food Microbiol* **23**: 277-294.

Baranyi, J., Roberts, T.A., and McClure, P. (1993) A non-autonomous differential equation to modelbacterial growth. *Food Microbiol* **10**: 43-59.

Kleindienst, S., Ramette, A., Amann, R., and Knittel, K. (2012) Distribution and *in situ* abundance of sulfate-reducing bacteria in diverse marine hydrocarbon seep sediments. *Environ Microbiol* **14**: 2689-2710.

Kleindienst, S., Herbst, F.-A., Stagars, M., von Netzer, F., von Bergen, M., Seifert, J. et al. (2014) Diverse sulfate-reducing bacteria of the *Desulfosarcina/Desulfococcus* clade are the key alkane degraders at marine seeps. *The ISME Journal* **8**: 2029-2044.

Loy, A., Lehner, A., Lee, N., Adamczyk, J., Meier, H., Ernst, J. et al. (2002) Oligonucleotide microarray for 16S rRNA gene-based detection of all recognized lineages of sulfate-reducing prokaryotes in the environment. *Appl Environ Microbiol* **68**: 5064-5081.

Lücker, S., Steger, D., Kjeldsen, K.U., MacGregor, B.J., Wagner, M., and Loy, A. (2007) Improved 16S rRNA-targeted probe set for analysis of sulfate-reducing bacteria by fluorescence in situ hybridization. *J Microbiol Methods* **69**: 523-528.

Macalady, J.L., Lyon, E.H., Koffman, B., Albertson, L.K., Meyer, K., Galdenzi, S., and Mariani, S. (2006) Dominant microbial populations in limestone-corroding stream biofilms, Frasassi cave system, Italy. *Appl Environ Microbiol* **72**: 5596-5609.

Manz, W., Eisenbrecher, M., Neu, T.R., and Szewzyk, U. (1998) Abundance and spatial organization of gram-negative sulfate-reducing bacteria in activated sludge investigated by in situ probing with specific 16S rRNA targeted oligonucleotides. *FEMS Microbiol Ecol* **25**: 43-61.

R: Core Team (2021). R: A language and environment for statistical computing. R foundation for statistical computing, Vienna, Austria. URL https://www.R-project.org/

Soetaert, K., Petzoldt, T., and Setzer, R.W. (2010) Solving differential equations in R: package deSolve. *J Stat Softw* **33**: 1 - 25.
